# Intestinal Source Control of Lipid Metabolism by Enzyme‐Probiotic Encapsulated, Spatiotemporal Crosslinked, and Small Intestine‐Adhesive Hydrogel Microspheres

**DOI:** 10.1002/advs.75362

**Published:** 2026-05-08

**Authors:** Xiaolin Wu, Weiwen Liang, Siqi He, Guangyuan Chen, Hui Zhou, Xinglong Wang, Yifei Li, Dingcai Wu, Bingna Zheng, Ruoxu Dou, Rongkang Huang

**Affiliations:** ^1^ Department of General Surgery The Fifth Affiliated Hospital Sun Yat‐sen University Zhuhai P. R. China; ^2^ Department of General Surgery (Thyroid Surgery) Guangdong Provincial Key Laboratory of Malignant Tumor Epigenetics and Gene Regulation Medical Research Center Sun Yat‐Sen Memorial Hospital Sun Yat‐Sen University Guangzhou P. R. China; ^3^ Department of Colorectal Surgery Foresea Life Insurance Guangzhou General Hospital Guangzhou P. R. China; ^4^ Colorectal Surgery Unit III Guangdong Institute of Gastroenterology Biomedical Innovation Center Guangdong Provincial Key Laboratory of Colorectal and Pelvic Floor Diseases The Sixth Affiliated Hospital Sun Yat‐sen University Guangzhou P. R. China; ^5^ Key Laboratory of Human Microbiome and Chronic Diseases, Ministry of Education Sun Yat‐sen University Guangzhou P. R. China; ^6^ PCFM Lab School of Chemistry Sun Yat‐sen University Guangzhou P. R. China; ^7^ The Eighth Affiliated Hospital Sun Yat‐sen University Shenzhen P. R. China

**Keywords:** akkermansia muciniphila, cholesterol oxidase, hydrogel microspheres, lipid metabolism, small intestinal mucosa adhesion

## Abstract

Probiotic therapy that targets fat absorption in the small intestine holds considerable potential for regulating lipid metabolism, yet it faces challenges related to spatiotemporal delivery and colonization efficiency. To address these issues, an enzyme‐probiotic biohybrid (AKK‐COD) has been developed and subsequently encapsulated in thiol‐modified alginate hydrogel microspheres. This therapeutic system enables sequential functions: intragastric protection, small intestinal adhesion, and probiotic release and colonization. The Ca^2+^‐crosslinked hydrogel maintains structural integrity under acidic gastric conditions, ensuring protection of probiotics and enzymes while facilitating timely gastric emptying. Upon reaching the small intestine, the microspheres adhere to the small intestinal mucosa via disulfide linkage, enhancing local retention. Concurrently, the gradual dissociation of ionic crosslinks within the hydrogel allows the release of AKK‐COD, which participates in the catalytic conversion of cholesterol, modulates lipid absorption pathways, and promotes microbial homeostasis. In vivo experiments demonstrate that this approach significantly alleviates high‐fat diet‐induced hypercholesterolemia, offering a safe and efficient strategy for lipid‐lowering therapies.

## Graphical abstract

A thiol‐modified alginate hydrogel microsphere‐encapsulated enzyme‐probiotic biohybrid (AKK‐COD) system has been developed to address the spatiotemporal delivery and colonization challenges of small intestine‐targeted probiotic for lipid metabolism regulation. The system exerts sequential functions of intragastric protection, small intestinal adhesion, and AKK‐COD release/colonization, catalyzing cholesterol conversion, modulating lipid absorption, and alleviating high‐fat diet‐induced hypercholesterolemia, thereby providing a safe and efficient lipid‐lowering strategy.

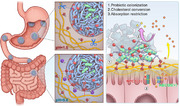

## 1 Introduction

Lipids, serving as essential structural building blocks and energy sources for cells, tissues, and organs, play a pivotal role in sustaining normal physiological functions [1]. However, chronic high‐fat diets frequently induce a cluster of metabolic abnormalities, including atherosclerosis, hepatic steatosis, insulin resistance, intestinal microecological imbalance, and obesity [2, 3]. These disorders collectively contribute to the pathogenesis of non‐alcoholic fatty liver disease, cardiovascular diseases, and other systemic complications, posing severe threats to human health and a significant socioeconomic burden [4]. The liver, as one of the core organs for lipid metabolism [5], undertakes the primary regulatory function of lipids after their absorption into the bloodstream, making it the target organ for traditional lipid‐lowering therapy in the clinic [6]. Nevertheless, long‐term medication often leads to liver damage, placing clinical treatment in a dilemma [2, 7, 8]. In parallel, the gastrointestinal tract, particularly the small intestine, serves as a major site for dietary lipid digestion and absorption due to its high density of absorption receptors and diverse intestinal microecosystem, establishing it as a critical alternative target for interventions [9]. In contrast to systemic post‐absorptive therapies that act on organs like the liver, regulating lipids at this pre‐absorptive stage within the intestine provides an inherent “source control” benefit. However, effectively harnessing this “source control” presents significant challenges, primarily due to the harsh and dynamic gastrointestinal environment which complicates the targeted delivery and sustained local action of therapeutic agents. Therefore, developing novel oral therapeutic approaches to intervene in lipid absorption in the small intestine has become a major trend in preventing and treating lipid metabolism disorders.

In recent years, non‐invasive therapeutic strategies like probiotic therapies have become an emerging field of human metabolic regulation [10, 11], thus demonstrating great potential in lipid modulation. It is reported that *Akkermansia* *muciniphila* (AKK) can reduce blood cholesterol and triglyceride levels and improve insulin sensitivity, thereby ameliorating chronic metabolic diseases such as fatty liver disease, hypercholesterolemia, and type 2 diabetes mellitus [10, 12, 13, 14]. However, oral probiotic delivery to the small intestine is constrained by complex physiological barriers, including the strongly acidic gastric environment, the action of proteolytic enzymes, and the “flushing effect” of small intestinal contents emptying, posing significant challenges to sufficient probiotic activity and long‐term colonization [15]. Conventional surface polymer coatings for probiotics delivery, such as calcium alginate and polydopamine, typically fail to meet the spatiotemporal delivery requirements of efficient gastric emptying and long‐term small intestinal colonization, resulting in suboptimal delivery efficacy. Specifically, efficient gastric emptying demands that probiotic coatings avoid excessive adhesion in the stomach, while long‐term small intestinal colonization requires strong bio‐adhesion at the small intestinal biological interface. This has long been considered difficult to achieve simultaneously in the same oral delivery material. Furthermore, although an enzyme (cholesterol oxidase) that directly catalyzes cholesterol conversion and is produced by a microbe has been discovered [16, 17], direct oral administration of the enzyme is also limited by the aforementioned physiological barriers, restricting its catalytic function in the early stage of intestinal absorption. Therefore, developing a probiotic treatment system with efficient gastric emptying, long‐term small intestinal colonization, and lipid‐lowering properties is crucial in opening new avenues for source regulation of lipid metabolism.

**FIGURE 1:**
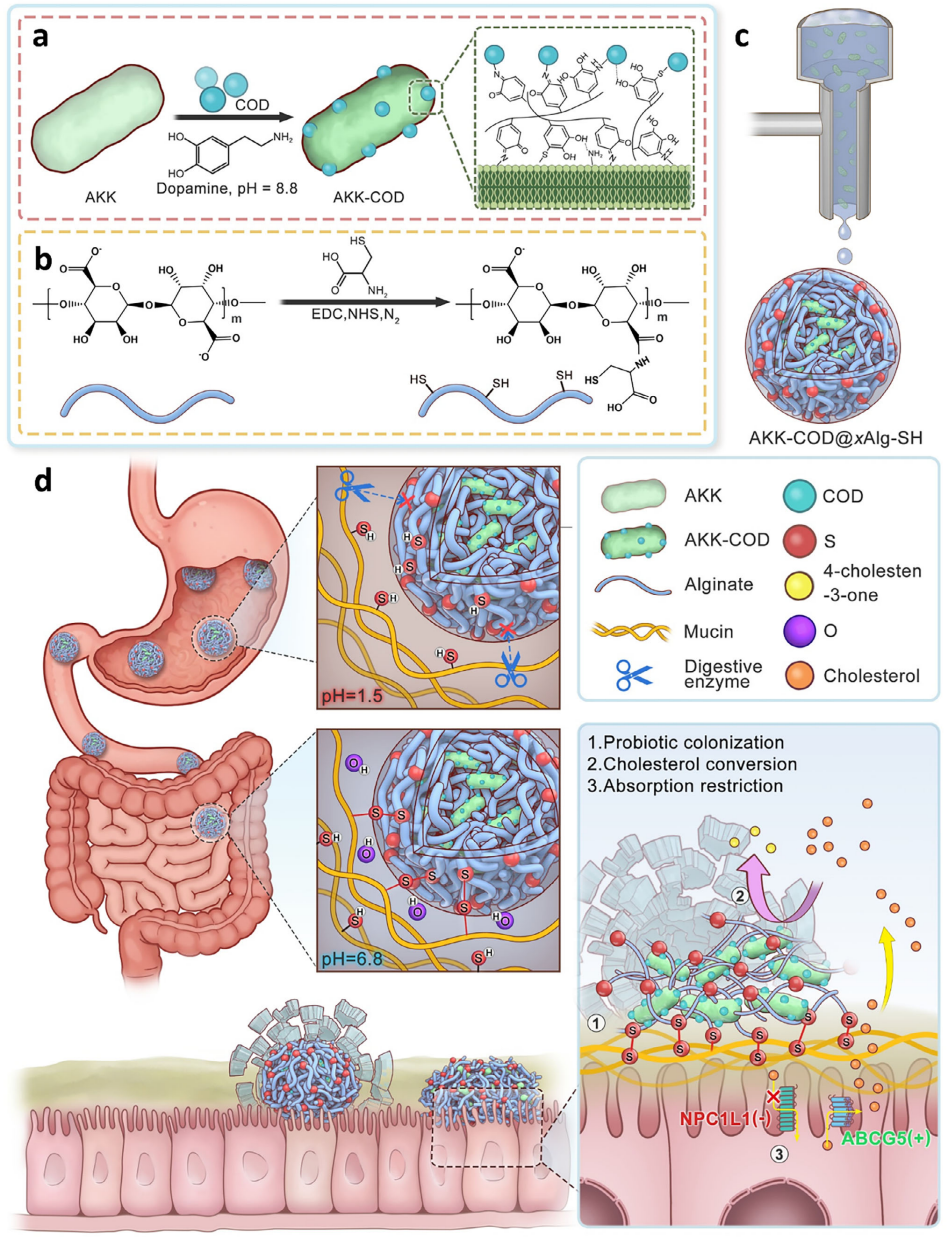
Schematic illustration of the preparation and therapeutic performances of AKK‐COD@*x*Alg‐SH. (a) Preparation of AKK‐COD by in situ self‐polymerization of dopamine. (b) Preparation of L‐cysteine modified sodium alginate. (c) Preparation of AKK‐COD@*x*Alg‐SH by using gas microfluidics and calcium ionic crosslinking techniques. (d) Oral delivery route of AKK‐COD@*x*Alg‐SH demonstrating sequential functions, including intragastric protection, small intestinal adhesion, and probiotics colonization, cholesterol conversion, and lipid absorption modulation.

Herein, by encapsulating AKK, which is recognized as a next‐generation probiotic, into the spatiotemporally controlled Ca^2+^‐crosslinked thiol‐modified alginate (*x*Alg‐SH) microspheres after chemical modification with cholesterol oxidase (COD), a biohybrid‐based “living” enzyme therapeutic system (denoted as AKK‐COD@*x*Alg‐SH) is obtained (Figure 1a–c). This system effectively overcomes physiological barriers involved in lipid absorption and regulation through a process of sequential intragastric protection and emptying, small intestinal adhesion, and subsequent probiotic release and colonization, thereby achieving lipid metabolism regulation (Figure 1d). The polydopamine (PDA) shell, serving as a covalent coupling medium between COD and AKK, can achieve stable loading of COD with high activity while ensuring viability of AKK. Benefiting from the structural stability of *x*Alg‐SH under acidic conditions, dual protection of probiotics and enzyme can be achieved during the gastric digestion stage. After AKK‐COD@*x*Alg‐SH enters the small intestine through gastrointestinal peristalsis, the *x*Alg‐SH shell can react with thiol groups in the small intestinal mucosa under neutral/weak alkaline conditions, facilitating the retention of AKK‐COD@*x*Alg‐SH. Subsequently, with the dissociation of Ca^2+^‐alginate crosslinks in *x*Alg‐SH, AKK‐COD is released into the small intestine. The COD component directly catalyzes the conversion of intraluminal cholesterol, while the AKK probiotic concurrently modulates lipid absorption pathways (e.g., downregulating NPC1L1 and upregulating ABCG5), collectively reducing lipid absorption into the bloodstream. Moreover, this composite system simultaneously repairs the intestinal barrier, and regulates microbial homeostasis within the small intestine. As demonstrated in high‐fat diet (HFD)‐fed mice, our AKK‐COD@*x*Alg‐SH can effectively attenuate diet‐induced hyperlipidemia and hepatic steatosis, and reverse intestinal barrier dysfunction. Therefore, our work provides new insights for the “source control” of various lipid metabolic disorders, offering broad implications for managing conditions like metabolic syndrome.

## 2 Results and Discussion

### 2.1 Preparation and Characterization of AKK‐COD@*x*Alg‐SH

**FIGURE 2:**
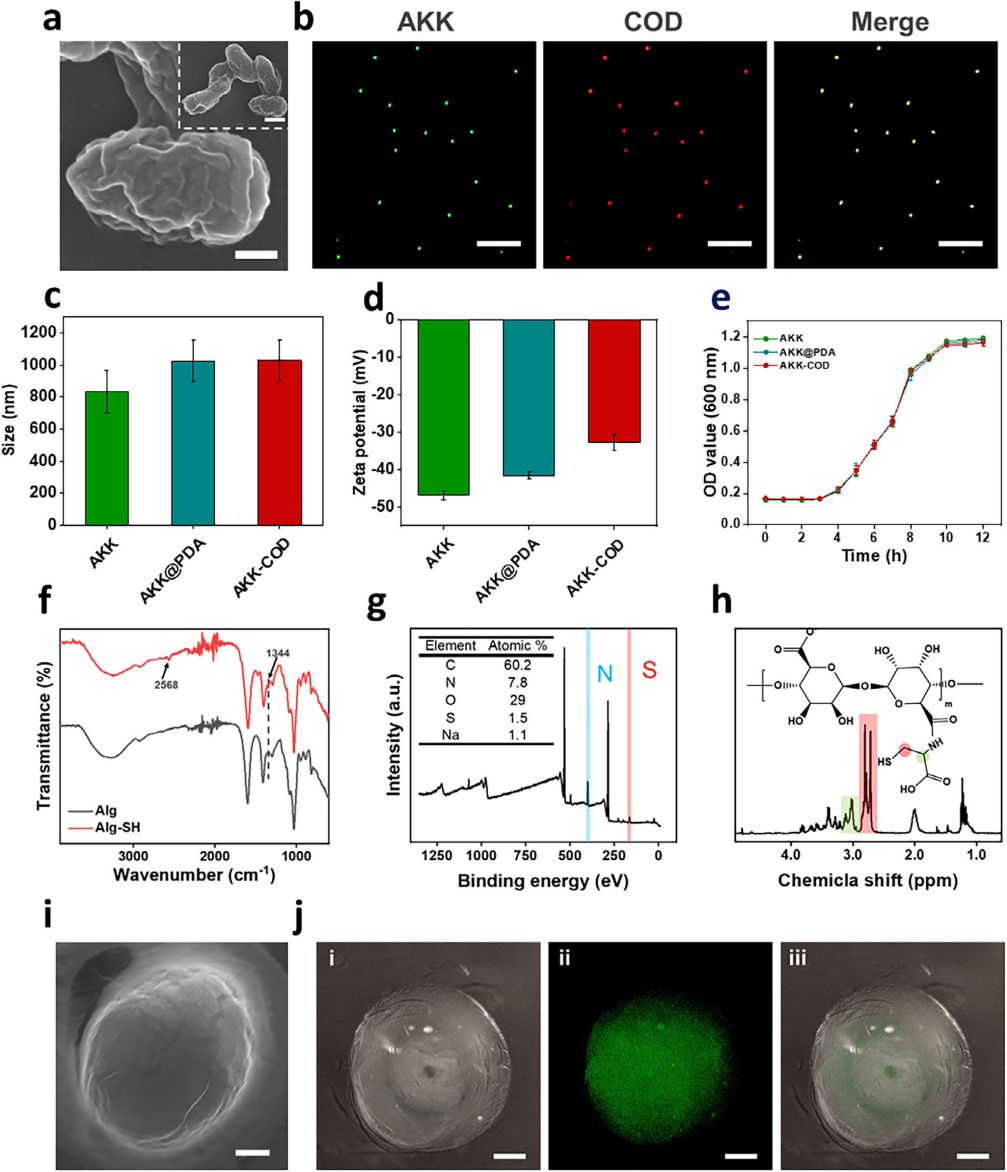
Characterization of AKK‐COD@*x*Alg‐SH. (a) SEM images of AKK‐COD. Scale bars: 150 and 250 nm (the inset). (b) Fluorescence microscope images of AKK‐COD, suggesting a large quantity of COD was covalently immobilized onto the surface of AKK. COD and AKK are labeled by Cy5.5 (red) and Syto9 (green), respectively. Scale bars: 10 µm. (c) Hydrodynamic sizes of AKK, AKK@PDA, and AKK‐COD based on DLS (*n*  =  3 independent samples; error bars = standard deviation (SD); the data are presented as mean ± SD). (d) Zeta potential results of AKK, AKK@PDA, and AKK‐COD (*n*  =  3 independent samples; the data are presented as mean ± SD). (e) Growth curves of AKK, AKK@PDA, and AKK‐COD (*n*  =  3 independent samples; data are presented as mean ± SD). (f) FT‐IR spectra of Alg and Alg‐SH. (g) XPS spectrum of Alg‐SH. (h) ^1^H NMR result of Alg‐SH. Scale bars: 2 mm. (i) SEM image of *x*Alg‐SH microsphere. Scale bar: 50 µm. (j) Fluorescence microscope images of AKK‐COD@*x*Alg‐SH with AKK being labeled by Syto9 (green): (i) image observed under bright‐field mode, (ii) image observed under fluorescence mode, (iii) merged image of (i) and (ii). Scale bars: 50 µm.

AKK, recognized for its beneficial effects on lipid metabolism [13], was selected as the “living” carrier for COD. COD‐modified AKK (AKK‐COD) was prepared via deposition of PDA under alkaline conditions (pH = 8.8) through self‐polymerization of dopamine, where COD was incorporated into PDA shell via covalent and hydrogen bonds (Figure 1a) [18, 19]. As shown in scanning electron microscopy (SEM) images, AKK‐COD preserves the elliptical morphology of unmodified AKK (Figure 2a; Figure S1). Fluorescence microscopy confirms the co‐localization of Cy5.5‐labeled COD and Syto9‐labeled AKK (Figure 2b), indicating successful loading of COD. Dynamic light scattering (DLS) reveals a slight increase in particle diameter of AKK@PDA (1027 nm) and AKK‐COD (1028 nm) compared to unmodified AKK (833 nm, Figure 2c; Figure S2). With the decoration of positively charged COD, the zeta potential of AKK‐COD (−32.7 mV) is higher than that of PDA‐coated AKK (AKK@PDA) (−41.5 mV, Figure 2d). According to the growth curves of AKK, AKK@PDA, and AKK‐COD, no significant difference is observed among the three groups (Figure 2e), indicating minimal impact on bacterial proliferation after COD loading.

To enhance gastric resistance and promote intestinal mucosal adhesion of AKK‐COD, *x*Alg‐SH microspheres, crosslinked by both Ca^2+^‐alginate ionic bond and reversible disulfide linkage, were introduced to encapsulate AKK‐COD for oral probiotic delivery. L‐cysteine is first modified to alginate chains via an amide reaction to produce thiol‐modified alginate (Alg‐SH). As shown in Fourier transform infrared (FT‐IR) spectra, the peaks at 1344 and 2568 cm^−1^ represent C─N and S─H stretching vibrations of amido bonds and thiol groups, respectively (Figure 2f), confirming the successful modification of L‐cysteine. X‐ray photoelectron spectroscopy (XPS) detects the characteristic S element in Alg‐SH with a 1.44% elemental content (Figure 2g). ^1^HNMR spectra further confirm the successful modification (Figure 2h). Subsequently, the solution of Alg‐SH (1% w/v) is added into calcium chloride solution (100 mm) by using a gas shearing method. Simultaneous formation of Ca^2+^‐Alg‐SH crosslinking leads to the *x*Alg‐SH microspheres. According to the image of microscope, *x*Alg‐SH microspheres demonstrate a mean diameter of 251 ± 17 µm (Figure S3). SEM reveals the spherical morphology of *x*Alg‐SH microspheres with a smooth surface appearance (Figure 2i). The interior of *x*Alg‐SH microsphere demonstrates a macro‐porous structure, and energy‐dispersive spectroscopy (EDS) further reveals the uniform distribution of S element in Alg‐SH (Figures S4 and S5). To evaluate the encapsulation status of AKK‐COD in *x*Alg‐SH microspheres, the Syto9‐labeled AKK‐COD uniformly distributes in *x*Alg‐SH according to the fluorescence microscopy (Figure 2j). Furthermore, cross‐sectional SEM images of the microspheres showing AKK‐COD inside the microspheres also confirm successful encapsulation (Figure S6). The encapsulation efficiency (EE) of AKK‐COD is 85.4% ± 2.7%, and the loading capacity (LC) of AKK‐COD is 6.23 ± 0.30 × 10^6^ CFU mg^−1^.

### 2.2 Protective Effect on Probiotics and Enzymatic Activity

**FIGURE 3:**
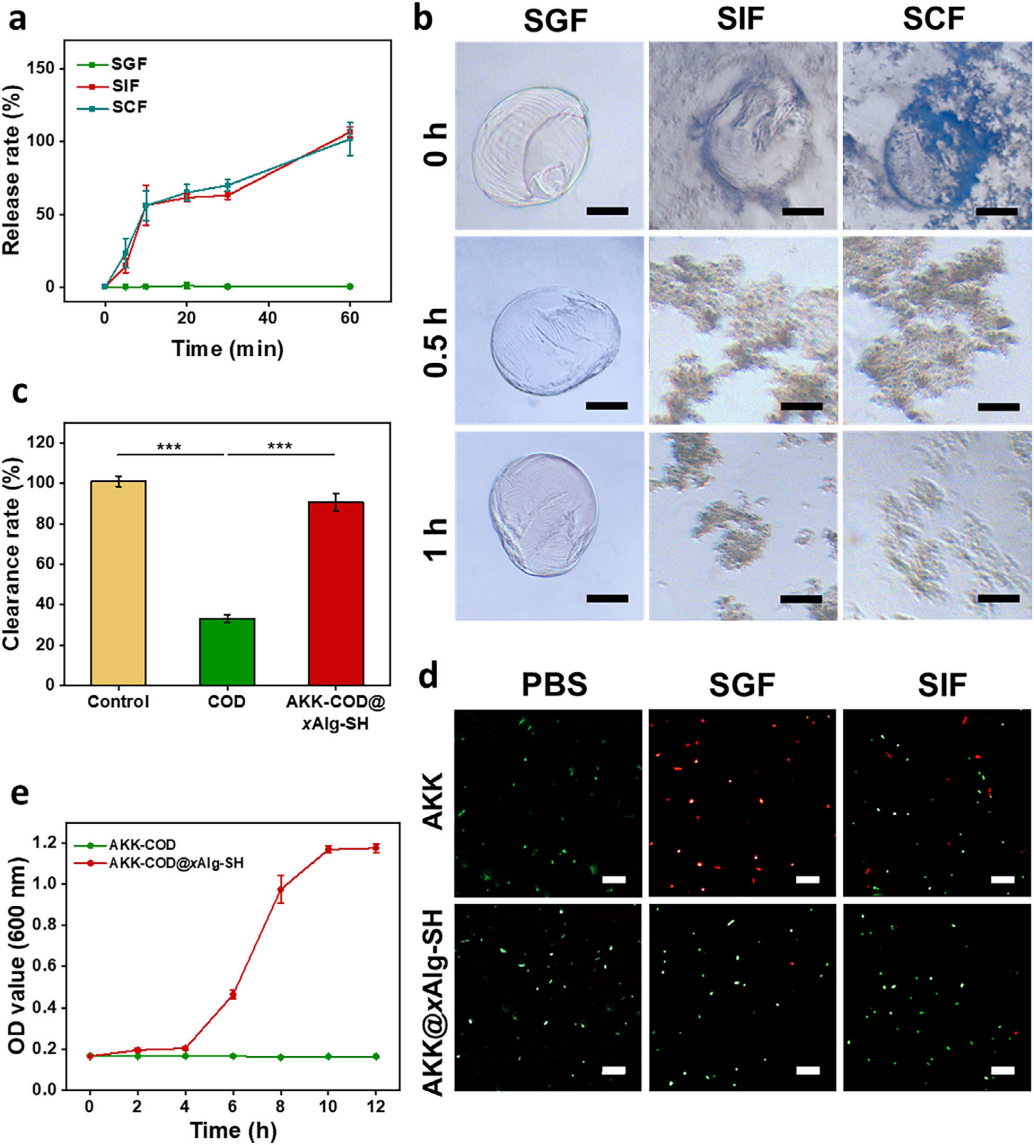
pH responsive behavior, catalytic performance, and gastric protective effect of AKK‐COD@*x*Alg‐SH. (a) Release profiles of AKK‐COD from *x*Alg‐SH microspheres in SGF, SIF, and SCF (*n*  =  3 independent samples; the data are presented as mean ± SD). (b) Morphological changes of AKK‐COD@*x*Alg‐SH microspheres in SGF, SIF, and SCF. Scale bars: 100 µm. (c) Catalytic efficiency comparison of AKK‐COD and AKK‐COD@*x*Alg‐SH after 6 h SGF treatment, the control group refers to the catalytic efficiency of AKK‐COD without SGF treatment (*n*  =  3 independent samples; the data are presented as mean ± SD; ANOVA followed by Tukey's multiple comparisons; ^***^ adjusted *p* < 0.001). (d) Live/dead staining images of AKK and AKK@*x*Alg‐SH after SGF treatment. Scale bars: 10 µm. (e) Growth curves of AKK‐COD and AKK‐COD@*x*Alg‐SH after SGF treatment (*n*  =  3 independent samples; the data are presented as mean ± SD).

To systematically evaluate the pH‐responsive release and self‐protective properties of AKK‐COD@*x*Alg‐SH, a series of in vitro assays were conducted under physiological simulation conditions. As shown in the pH‐responsive release assays, *x*Alg‐SH microspheres remain stable in simulated gastric fluid (SGF, pH 1.5) and no release of AKK‐COD is observed, while *x*Alg‐SH microspheres disintegrate rapidly in simulated intestinal fluid (SIF, pH 6.8) and simulated colonic fluid (SCF, pH 7.2) with over 50% release of AKK‐COD within 10 min (Figure 3a). The morphological changes in different conditions at 0, 30, and 60 min are observed in Figure 3b. The microspheres exhibit no obvious morphological changes in SGF, whereas they degrade rapidly in SIF and SCF, indicating the pH‐responsive characteristic of *x*Alg‐SH.

To ensure the catalytic function of AKK‐COD in the small intestine, the in vitro cholesterol clearance assays were carried out to verify the catalytic activity of AKK‐COD in SIF at 37°C, which was tested at 24 h intervals over 7 days. As shown in Figure S7, AKK‐COD maintains high catalytic efficiency (> 70%) throughout the testing period, which is critical for ensuring continuous modulation of cholesterol metabolism.

The gastric acid protection provided by *x*Alg was assessed by measuring the catalytic activity of COD and the viability of AKK in an SGF environment. The gastric acid protection assay reveals that after exposure to SGF, the AKK‐COD group retains only about 33.1% of its catalytic efficiency (Figure 3c). In sharp contrast, the AKK‐COD@*x*Alg‐SH group maintains 90.7% of its catalytic efficiency (Figure 3c), demonstrating that *x*Alg‐SH can protect the enzymatic activity of COD from gastric fluid damage. Live/dead staining images visually confirm the higher viability of AKK@*x*Alg‐SH compared to unprotected AKK after SGF treatment (Figure 3d), demonstrating the critical role of *x*Alg‐SH microspheres in maintaining microbial and enzymatic activity. The growth curves of AKK‐COD protected by *x*Alg‐SH microspheres indicate that AKK‐COD@*x*Alg‐SH can maintain normal proliferation in SGF, while unprotected AKK‐COD shows severely impaired growth (Figure 3e). The above results demonstrate that *x*Alg‐SH microspheres effectively prevent premature release of AKK‐COD and maintain their activities in the stomach while ensuring effective delivery of AKK‐COD to the intestinal tract.

### 2.3 On‐Demand Gastric Emptying and Small Intestinal Colonization

**FIGURE 4:**
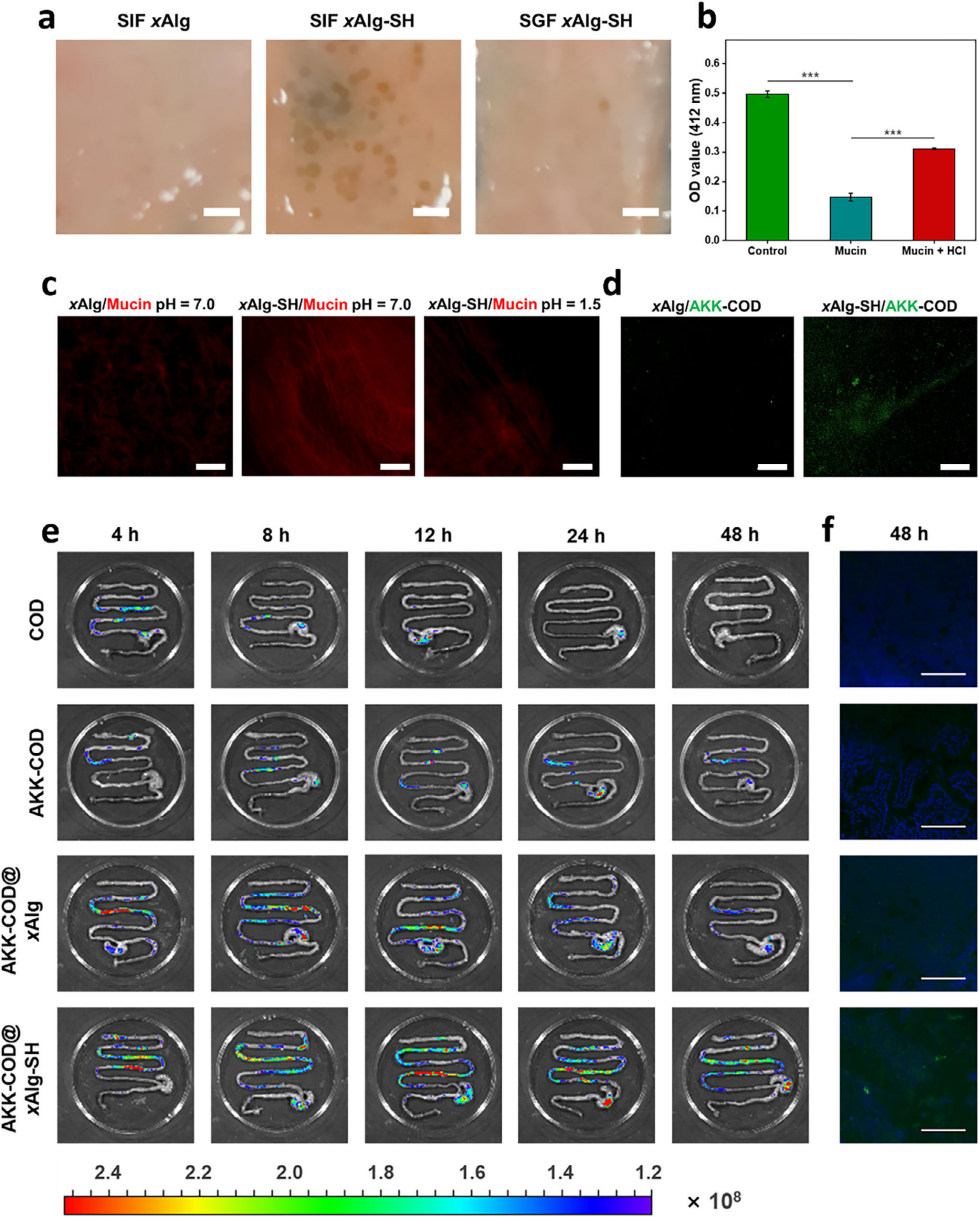
Small intestinal adhesion and probiotics colonization. (a) Images of interaction between rat intestine and *x*Alg‐SH microspheres. Scale bars: 1 mm. (b) Semiquantitative test for reactive thiol groups of *x*Alg‐SH under different conditions (*n*  =  3 independent samples; the data are presented as mean ± SD; ANOVA followed by Tukey's multiple comparisons; ^***^ adjusted *p* < 0.001). (c) Fluorescence microscopy results of Cy5.5‐labeled mucin incubated with *x*Alg‐SH and *x*Alg membranes for 30 min followed by washing. Scale bars: 100 µm. (d) Fluorescence microscopy results of Syto9‐labeled AKK‐COD incubated with *x*Alg‐SH and *x*Alg membranes for 30 min followed by washing. Scale bars: 100 µm. (e,f) In vivo fluorescence imaging results (e) and microscopic fluorescence images (f) of FITC‐labeled COD in C57BL/6 mice. Scale bars: 200 µm.

High bio‐adhesion is indispensable for long‐term small intestinal colonization and full exertion of the cholesterol‐catalyzing function, whereas low gastric adhesion is critical for efficient gastric emptying, thus leading to a contradictory requirement that remains a major challenge for oral probiotic delivery materials. In an in vitro adhesion assay using rat small intestine, the microspheres were incubated with intestinal mucosa for 2 min under different simulated conditions. It is found that in SIF, almost no *x*Alg microspheres remain on the intestinal tissue, whereas a number of *x*Alg‐SH microspheres are retained on the intestinal tissue (Figure 4a). In contrast, under simulated gastric fluid conditions, the retention of microspheres is markedly reduced (Figure 4a). This suggests that *x*Alg‐SH microspheres may bind more tightly under neutral conditions than under acidic conditions. Total thiol assay kit with 5,5’‐dithiobis‐(2‐nitrobenzoic acid) (DTNB) was used to evaluate the thiol groups of *x*Alg‐SH microspheres under different environments. As shown in Figure 4b, after a 30 min incubation with mucin (the characteristic content of intestinal mucus), the OD_412_ of *x*Alg‐SH decreases drastically compared with the control group, indicating that the thiol groups are decreased after the reaction between *x*Alg‐SH and mucin. In contrast, the OD_412_ of the microspheres incubated with mucin at low pH (1.5) shows a slight decrease (Figure 4b), indicating that disulfide bond formation is inhibited under acidic conditions. These results suggest that *x*Alg‐SH microspheres achieve selective adhesion in the small intestine through pH‐dependent changes in thiol‐mediated binding affinity along the gastrointestinal tract.

To examine the adhesion between microspheres and mucin or microbe, *x*Alg‐SH and *x*Alg hydrogel membranes were prepared in the well plates. After incubation with Cy5.5‐labeled mucin and subsequent washing, the fluorescence intensity of the *x*Alg‐SH/Mucin (pH = 7.0) group is higher than that of the *x*Alg/Mucin (pH = 7.0) and *x*Alg‐SH/Mucin (pH = 1.5) groups (Figure 4c; Figure S8). In addition, the Syto9‐labeled AKK‐COD shows stronger retention on *x*Alg‐SH membrane than on *x*Alg membrane (Figure 4d; Figure S9). Isothermal titration calorimetry (ITC) was further employed to investigate the interactions of Alg and Alg‐SH with both mucin and AKK‐COD. Alg‐SH and Alg interact exothermically with both mucin and AKK‐COD. The association constant (Ka) of Alg‐SH with mucin is 8.9 × 10^5^ m ^−1^, which is higher than that of Alg with mucin (4.0 × 10^3^ m ^−1^, Figure S10a–c,f), indicating the formation of stronger binding interactions (e.g., disulfide bond). Similar trends are discovered in the reaction systems of AKK‐COD with Alg (9.6 × 10^3^ m ^−1^) and AKK‐COD with Alg‐SH (2.7 × 10^5^ m ^−1^, Figure S10d,e,g). These results demonstrate the potential ability of *x*Alg‐SH to act as a bridge between the mucin and microbe.

To verify the interactions of *x*Alg‐SH with mucin and intestine from the mechanical aspect, in vitro adhesion force measurements were performed. *x*Alg‐SH membrane exhibits nearly double the maximum detachment forces (MDFs) of *x*Alg, with values of 1.07 vs. 0.55 N on 2% mucin layer, and 0.39 vs. 0.22 N on rat intestinal mucosa, respectively (Figure S11). In vivo fluorescence imaging was conducted using fluorescein 5‐isothiocyanate (FITC)‐labeled COD to validate the role of *x*Alg‐SH in assisting the colonization of the active component. In the COD group, the fluorescence signal has been almost completely cleared from the small intestine and has entered the colon by 12 h (Figure 4e). In the AKK‐COD and AKK‐COD@*x*Alg groups, the signal is predominantly distributed in the colon by 24 h (Figure 4e). In contrast, the AKK‐COD@*x*Alg‐SH group shows substantial fluorescence distribution in the small intestine even at 48 h (Figure 4e). Histological section analysis of small intestinal tissue at 48 h further confirms the abundant presence of fluorescence‐labeled COD within the intestinal lumen in the AKK‐COD@*x*Alg‐SH group (Figure 4f). Therefore, these results indicate that the thiol groups on *x*Alg‐SH can remain stable under gastric pH conditions, thereby minimizing the adhesion between AKK‐COD@*x*Alg‐SH and the gastric mucosa. In addition, they can also react with probiotics and the mucus layer in the small intestine by forming disulfide bonds, thus enhancing interactions between AKK‐COD and intestinal mucus to facilitate mucosal adhesion and probiotics colonization.

### 2.4 Biosafety of AKK‐COD@*x*Alg‐SH In Vitro

Multiple in vitro biocompatibility assays were performed to evaluate the biosafety of our AKK‐COD@*x*Alg‐SH microspheres system. Caco‐2 and RAW264.7 cells were incubated with extracts from COD and AKK‐COD@*x*Alg‐SH hydrogel microspheres for 1, 2, and 3 days, enabling qualitative and quantitative assessments of the biocompatibility. As shown in the live/dead staining assay of Caco‐2 and RAW264.7 cells, both the COD and AKK‐COD@*x*Alg‐SH groups exhibit biocompatibility comparable to that in the control group (Figure S12a,c). Cell Counting Kit‐8 (CCK‐8) results further confirm the favorable biocompatibility of AKK‐COD@*x*Alg‐SH over 3 days (Figure S12b,d). Additionally, the biocompatibility of *x*Alg‐SH as a bacterial delivery carrier was tested (Figure S12e). Co‐culture experiments with AKK reveal that neither *x*Alg nor *x*Alg‐SH significantly affects bacterial proliferation within 24 h, indicating that *x*Alg‐SH is an excellent carrier with minimal impact on the viability of delivered probiotics. These results indicate that AKK‐COD@*x*Alg‐SH microspheres have excellent biocompatibility.

### 2.5 Therapeutic Efficacy and Mechanism in Hypercholesterolemia Model

**FIGURE 5:**
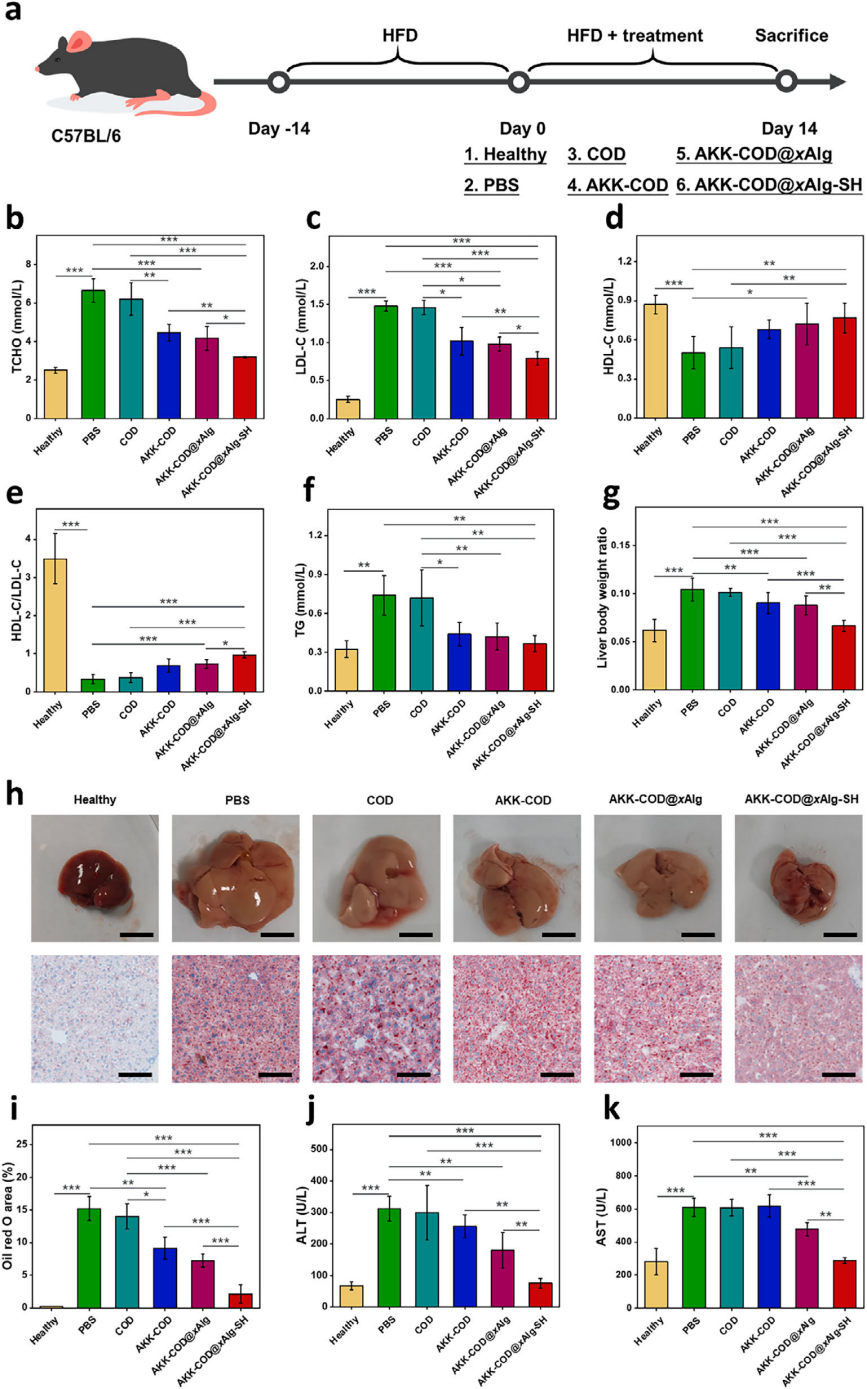
Therapeutic efficacy of AKK‐COD@*x*Alg‐SH in hypercholesterolemia models. (a) Schematic diagram of high‐fat diet modeling, grouping, and treatment in C57BL/6 mice. (b–f) Quantitative comparisons of total cholesterol (b), high‐density lipoprotein cholesterol (c), low‐density lipoprotein cholesterol (d), HDL‐C/LDL‐C ratio (e), and triglycerides (f) across groups after treatment. (g) Analysis of liver weight/body weight ratio after treatment. (h) Digital photos and Oil Red O staining of fatty liver status across groups after treatment. Scale bars: 1 cm (upper row) and 100 µm (lower row). (i) Quantitative analysis of liver Oil Red O staining after treatment (*n*  =  3 independent samples; the data are presented as mean ± SD; ANOVA followed by Tukey's multiple comparisons; ^*^ adjusted *p* < 0.05, ^**^ adjusted *p* < 0.01, ^***^ adjusted *p* < 0.001). (j,k) Quantitative comparisons of alanine aminotransferase (j) and aspartate aminotransferase (k) after treatment (*n*  =  3 independent samples; the data are presented as mean ± SD; ANOVA followed by Tukey's multiple comparisons; ^**^ adjusted *p* < 0.01, ^***^ adjusted *p* < 0.001).

To evaluate the therapeutic efficacy of AKK‐COD@*x*Alg‐SH in hypercholesterolemia, a mouse model was established by feeding C57BL/6 mice a high‐fat diet. After 2 weeks, the mice were gavaged once daily with PBS, COD, or formulations containing AKK‐COD (i.e., AKK‐COD, AKK‐COD@*x*Alg, and AKK‐COD@*x*Alg‐SH) at doses equivalent to 2 U d^−1^ or 1 × 10^9^ CFU d^−1^ for an additional 2 weeks, while the healthy group was fed standard rodent chow and water (Figure 5a). Blood lipid levels were measured at the end of the experiment. Mice in the PBS group exhibit elevated levels of total cholesterol (TCHO), low‐density lipoprotein cholesterol (LDL‐C), and triglycerides (TG) along with reduced high‐density lipoprotein cholesterol (HDL‐C) and HDL‐C/LDL‐C ratios (Figure 5b–f), suggesting the hypercholesterolemia model was successfully established. In comparison, administration of formulations containing AKK‐COD decreases TCHO, LDL‐C, and TG levels while increasing HDL‐C level and HDL‐C/LDL‐C ratios, particularly in the AKK‐COD@*x*Alg‐SH group (Figure 5b–f). Specifically, the AKK‐COD formulation therapy groups show a significant decrease in levels of TCHO, LDL‐C, and TG compared with the COD group, which may indicate a key role of AKK in modulating lipid metabolism (Figure 5b,c,f). Moreover, relative to the AKK‐COD@*x*Alg group, the AKK‐COD@*x*Alg‐SH group exhibits marked improvements in TCHO, LDL‐C, and HDL‐C/LDL‐C (Figure 5b,c,e), suggesting that small intestinal‐assisted colonization provided by *x*Alg‐SH is essential for enabling AKK‐COD to efficiently regulate lipid metabolism.

Fatty liver status was also used to assess the improvement of hyperlipidemia. In the comparison of liver‐to‐body weight ratio, the AKK‐COD@*x*Alg‐SH group demonstrates the best therapeutic outcome among all treatment groups, with no significant difference from the healthy control group (Figure 5g). Visual assessment of liver morphology further confirmed the therapeutic superiority of the AKK‐COD@*x*Alg‐SH formulation. Notably, the AKK‐COD@*x*Alg‐SH group exhibits a markedly reduced liver size compared to both the PBS and COD groups (Figure 5h), underscoring the critical contribution of AKK to protecting the liver. Furthermore, Oil Red O staining was employed to evaluate lipid deposition in the liver. Similarly, treatments with AKK‐COD formulations lead to a significant decrease in lipid deposition compared with the COD group, and the AKK‐COD@*x*Alg‐SH group exhibits superior efficacy relative to the AKK‐COD@*x*Alg group (Figure 5h,i). These results further suggest the lipid metabolism‑regulating function of AKK, as well as the potentially critical role of *x*Alg‑SH in the delivery of the probiotic‐enzyme biohybrid system. Markers of liver damage including alanine aminotransferase (ALT) and aspartate aminotransferase (AST) were assessed. The AKK‐COD@*x*Alg‐SH group exhibits less liver function injury during cholesterol‐lowering treatment (Figure 5j,k). Histological analysis by hematoxylin‐eosin (HE) staining shows no significant damage in major organs (heart, liver, spleen, lung, and kidney) and no obvious inflammation in gastrointestinal tissues (stomach, small intestine, and colon) (Figure S13), indicating excellent in vivo biosafety of AKK‐COD@*x*Alg‐SH. These results indicate that AKK‐COD@*x*Alg‐SH can regulate lipid metabolism without liver and systemic toxicity. Compared with traditional lipid‐lowering strategies represented by statins that mainly act on the liver to inhibit cholesterol synthesis, as well as ezetimibe that merely inhibits intestinal cholesterol absorption, our system performs “source control” directly at the intestinal absorption stage before lipids enter the bloodstream, thus avoiding the potential liver metabolism burden and side effects associated with systemic drugs. More importantly, this strategy integrates catalytic decomposition, transport regulation, and intestinal microecology repair, which is distinct from single‐target clinical drugs.

**FIGURE 6:**
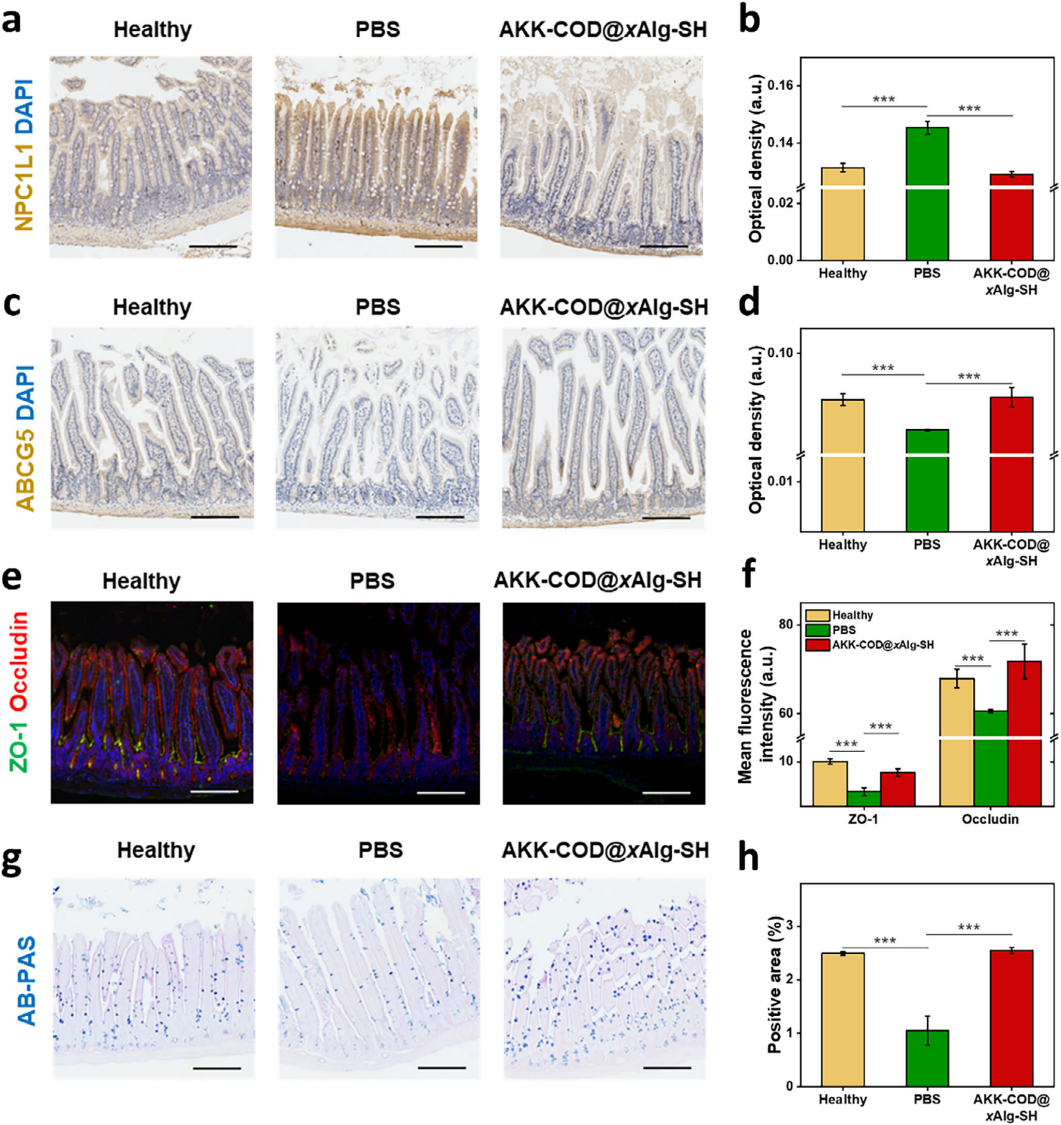
Exploration of the mechanisms of interaction between AKK‐COD@*x*Alg‐SH and intestinal epithelial. (a,b) Immunohistochemical staining images (a) and quantitative analysis (b) of NPC1L1 protein in the small intestine. (c,d) Immunohistochemical staining images (c) and quantitative analysis (d) of ABCG5 protein in the small intestine. (e,f) Immunofluorescence staining images (e) and quantitative analysis (f) of ZO‐1 (green) and Occludin (red) in the small intestine. (g,h) AB‐PAS staining images (g) and quantitative analysis (h) of goblet cells in the small intestine. Scale bars: 200 µm (a,c,e,g). *n*  =  3 independent samples; the data are presented as mean ± SD; ANOVA followed by Tukey's multiple comparisons; ^***^ adjusted *p* < 0.001.

To further explore the lipid‐lowering mechanism of AKK‐COD@*x*Alg‐SH, changes in fecal cholesterol levels were measured. Fecal cholesterol level shows no significant change in the AKK‐COD@*x*Alg‐SH group, whereas that in the PBS group is significantly increased (Figure S14). Fecal cholesterol levels in high‐fat diet‐fed mice increase with dietary cholesterol content [20], whereas the reduced fecal cholesterol observed in the AKK‐COD@*x*Alg‐SH group may be attributed to the conversion of unabsorbed intestinal cholesterol into 4‐cholesten‐3‐one by COD. Subsequent immunohistochemical staining (Figure 6a,c) and quantitative analysis (Figure 6b,d) reveal the downregulated Niemann‐Pick C1‐Like 1 (NPC1L1) and upregulated ATP‐Binding Cassette Sub‐Family G Member 5 (ABCG5) in the AKK‐COD@*x*Alg‐SH group. As a critical transporter for small intestinal cholesterol absorption [9, 21], downregulation of NPC1L1 reduces cholesterol uptake, while upregulation of ABCG5 promotes cholesterol efflux from intestinal epithelial cells [22]. Propionate produced by AKK in the gut may downregulate NPC1L1 expression by modulating the local immune environment [23]; moreover, the presence of AKK can stimulate ABCG5/8 transporter and decrease NPC1L1 levels via nuclear factor‐kappa B (NF‐κB) [12]. Additionally, COD degrades accumulated luminal cholesterol into 4‐cholesten‐3‐one in the small intestine [24], reducing cholesterol transport from the intestinal lumen to epithelial cells and collectively addressing hypercholesterolemia. Therefore, AKK‐COD@*x*Alg‐SH can significantly improve hypercholesterolemia by inhibiting small intestinal epithelial cholesterol absorption, enhancing reverse cholesterol transport, and reducing intestinal cholesterol levels via catalysis.

### 2.6 Reshaping of Intestinal Barrier Function and Intestinal Microbiota Balance

Intestinal barrier function and gut microbiota dysregulation are closely linked to the development of lipid metabolism disorders [25]. The effect of AKK‐COD@*x*Alg‐SH on intestinal barrier function was investigated. Immunofluorescence studies (Figure 6e,f) show lower Zonula Occludens‐1 (ZO‐1) and occludin expression in the PBS group compared to the healthy group, indicating barrier dysfunction induced by high‐fat diet. In contrast, significant upregulation of ZO‐1 and occludin expression is revealed in the AKK‐COD@*x*Alg‐SH group (Figure 6e,f). Quantitative analyses of goblet cells via Alcian Blue and Periodic Acid‐Schiff (AB‐PAS) staining further confirm that AKK‐COD@*x*Alg‐SH can reverse high‐fat diet‐induced intestinal barrier damage (Figure 6g,h).

**FIGURE 7:**
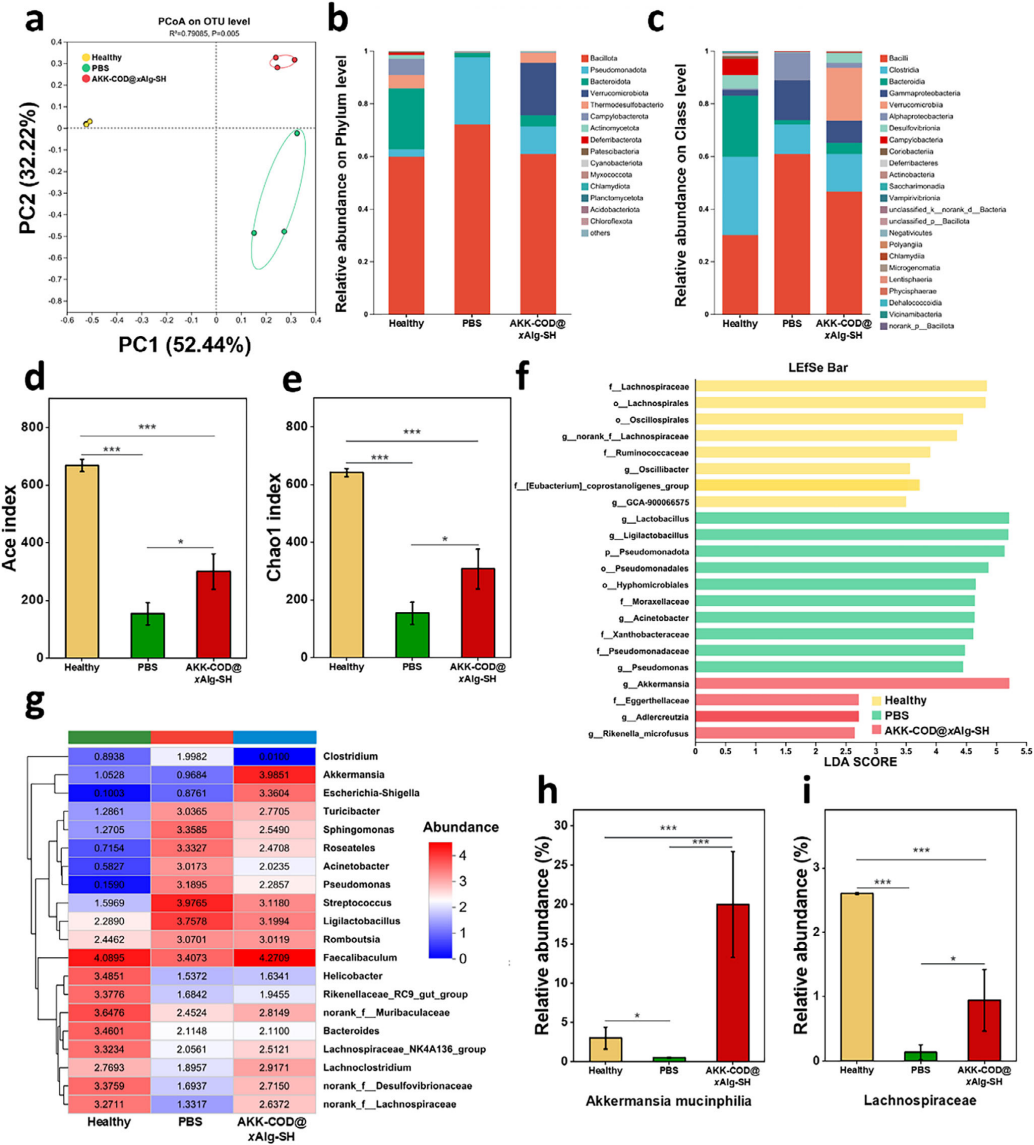
16S rRNA sequencing analysis of the effect of AKK‐COD@*x*Alg‐SH on gut microbiota. (a) PCA showing β‐diversity of the gut microbiota (*n*  =  3 independent samples). (b) Stacked bar chart of relative species abundances at the phylum level across different treatment groups. (c) Stacked bar chart of relative species abundances at the class level across different treatment groups. (d,e) Comparison of alpha diversity (Ace index [d] and Chao index [e]) across different treatment groups. (f) LDA identifying species with significant contributions to intergroup differences. (g) Heatmap of relative species abundances at the genus level across different treatment groups. (h) Comparison of *Akkermansia* abundances across different treatment groups (*n*  =  3 independent samples; the data are presented as mean ± SD; ANOVA followed by Tukey's multiple comparisons; ^*^ adjusted *p* < 0.05, ^***^ adjusted *p* < 0.001). (i) Comparison of *Lachnospiraceae* abundances across different treatment groups (*n*  =  3 independent samples; the data are presented as mean ± SD; ANOVA followed by Tukey's multiple comparisons; ^*^ adjusted *p* < 0.05, ^***^ adjusted *p* < 0.001).

Furthermore, 16S rRNA sequencing was employed to analyze the intestinal bacterial composition in mouse intestinal content. As shown in Figure 7a, principal component analysis (PCA) detects differences in community richness among the three groups (Healthy, PBS, and AKK‐COD@*x*Alg‐SH). Samples with more similar species compositions are closer in the 2D coordinate plot (Figure 7a), indicating that AKK‐COD@*x*Alg‐SH treatment alters the microbial community composition in high‐fat diet‐fed mice at the operational taxonomic units (OTU) level. The microbial composition at the phylum and class levels across treatment groups was also tested. The PBS group shows increased abundances of *Firmicutes* and *Pseudomonas*, as well as decreased *Bacteroidota* (Figure 7b,c). Treatment with AKK‐COD@*x*Alg‐SH partially reverses these high‐fat diet‐induced changes and increases the proportion of *Verrucomicrobiota* (Figure 7b,c). As the *Firmicutes/Bacteroidetes* ratio is a biomarker indicating obesity phenotypes [26], it was compared across groups. As shown in Figure S15, the *Firmicutes/Bacteroidetes* ratio is significantly elevated in the PBS group but reversed following AKK‐COD@*x*Alg‐SH treatment. Alpha diversity comparisons reveal that AKK‐COD@*x*Alg‐SH enhances community richness in the mouse small intestine (Figure 7d,e). Linear discriminant analysis (LDA) was performed to identify taxonomically distinct groups. The bar chart of LDA scores shows that *Akkermansia* is the characteristic species of the AKK‐COD@*x*Alg‐SH group (Figure 7f). A heatmap of microbial abundance at the genus level demonstrates higher *Akkermansia* levels in the AKK‐COD@*x*Alg‐SH group (Figure 7g), indicating enhanced enrichment of AKK in the small intestine. Kruskal‐Wallis test analysis of metabolism‐related bacterial species shows increased *Akkermansia muciniphila* in the AKK‐COD@*x*Alg‐SH group (Figure 7h), along with elevated levels of *Lachnospiraceae* (negatively associated with insulin resistance, Figure 7i) [27, 28] and *Faecalibaculum* (regulating intestinal epithelial homeostasis, Figure S16) [29]. These results suggest that AKK‐COD@*x*Alg‐SH can facilitate the restoration of intestinal mucosal barrier and reestablish the balance of gut microecology.

## 3 Conclusion

In summary, we have developed a novel biohybrid therapeutic system (AKK‐COD@*x*Alg‐SH) for source control of metabolic diseases via non‐invasive oral delivery. The porous, thiol‐modified alginate microsphere, crosslinked by both Ca^2^^+^ ions and disulfide bonds, serves as a spatiotemporally programmable carrier. This architecture maintains structural stability in the gastric environment due to acid‐resistant Ca^2^^+^ crosslinks, and facilitates pH‐triggered adhesion and dissociation in the intestinal tract through thiol‐disulfide exchange with mucin at neutral pH, thereby achieving sequential release in the target organ. Beyond precise delivery, the therapeutic efficacy is attributed to a synergistic biohybrid construct. The co‐localization of COD and AKK at the intestinal site enables concurrent functions: COD directly converts dietary cholesterol into 4‐cholesten‐3‐one, while the colonizing AKK downregulates the cholesterol transporter NPC1L1 and upregulates the efflux transporter ABCG5, thereby blocking absorption of lipids. Furthermore, sustained release and colonization of AKK restore gut ecological balance, enrich beneficial taxa (e.g., *Lachnospiraceae*), and repair the intestinal barrier via upregulation of tight junction proteins (ZO‐1, Occludin). In a murine model of diet‐induced hypercholesterolemia, our AKK‐COD@*x*Alg‐SH can significantly reduce serum cholesterol and triglycerides, attenuate hepatic steatosis, and exhibit excellent biocompatibility. Collectively, this work establishes a design rationale for integrating functional materials with living microbes, offering a versatile platform for oral delivery and intervention in complex metabolic disorders.

## 4 Experimental Section

The Experimental Section is available in the Supporting Information.

## Ethics Statement

All experiments complied with international guidelines, and animal studies were approved by the Institutional Animal Care and Use Committee of Sun Yat‐sen University (License No: 2024000290).

## Conflicts of Interest

The authors declare no conflicts of interest.

## Supporting information




**Supporting File**: advs75362‐sup‐0001‐SuppMat.docx.

## Data Availability

Data supporting the results of this study are available in the paper and its Supplementary Information. All data underlying this study are accessible from the corresponding authors upon request.
