# Supplementary material for: Intestinal Source Control of Lipid Metabolism by Enzyme‐Probiotic Encapsulated, Spatiotemporal Crosslinked, and Small Intestine‐Adhesive Hydrogel Microspheres
Source: Adv Sci (Weinh). 2026 May 8;13(39):e75362. doi: 10.1002/advs.75362 (PMC13335443; doi:10.1002/advs.75362)
Supplement: Supplementary file 1 — Supporting File: advs75362‐sup‐0001‐SuppMat.docx. [file ADVS-13-e75362-s001.docx]

**Intestinal Source Control of Lipid Metabolism by Enzyme-Probiotic Encapsulated, Spatiotemporally Crosslinked, and Small Intestine-Adhesive Hydrogel Microspheres**

Xiaolin Wu^1^, Weiwen Liang^2^, Siqi He^3^, Guangyuan Chen^4,5^, Hui Zhou^6^, Xinglong Wang^6^, Yifei Li^4,5^, Dingcai Wu^6,*^, Bingna Zheng^7,*^, Ruoxu Dou^3,*^, Rongkang Huang^4,5,*^

^1^Department of General Surgery, The Fifth Affiliated Hospital, Sun Yat-sen University, Zhuhai, P. R. China

^2^Department of General Surgery (Thyroid Surgery), Guangdong Provincial Key Laboratory of Malignant Tumor Epigenetics and Gene Regulation, Medical Research Center, Sun Yat-Sen Memorial Hospital, Sun Yat-Sen University, Guangzhou, P. R. China

^3^Department of Colorectal Surgery, Foresea Life Insurance Guangzhou General Hospital, Guangzhou, P. R. China

^4^Colorectal Surgery Unit III, Guangdong Institute of Gastroenterology, Biomedical Innovation Center, Guangdong Provincial Key Laboratory of Colorectal and Pelvic Floor Diseases, The Sixth Affiliated Hospital, Sun Yat-sen University, Guangzhou, P. R. China

^5^Key Laboratory of Human Microbiome and Chronic Diseases, Ministry of Education, Sun Yat-sen University, Guangzhou, P. R. China

^6^PCFM Lab, School of Chemistry, Sun Yat-sen University, Guangzhou, P. R. China

^7^The Eighth Affiliated Hospital, Sun Yat-sen University, Shenzhen, P. R. China

**Materials and Methods**

**Materials**

Sodium alginate (Alg), calcium chloride dihydrate powder, 1-ethyl-(3-dimethylaminopropyl) carbodiimide (EDC), N-hydroxy succinimide (NHS), cholesterol oxidase (COD), dopamine hydrochloride, Cy5.5-NHS, mucin, acetone, and isopropanol were purchased from Shanghai Aladdin Biochemical Technology Co., Ltd. (China). Cholesterol and L-cysteine were purchased from Shanghai Macklin Biochemical Technology Co., Ltd. (China). Phosphate-buffered saline (PBS) buffer (pH = 7.2 ~ 7.4), saline, ezetimibe, Ellman’s reagent, Cell Counting Kit-8 (CCK-8), and Calcein acetoxymethyl ester (calcein-AM) staining kit were purchased from Dalian meilunbio Biotechnology Co., Ltd. (China). Simulated gastric fluid (SGF), simulated intestinal fluid (SIF), and simulated colonic fluid (SCF) were purchased from Shanghai MesGen Biotech Co., Ltd. (China). Tris-HCl buffer (pH = 8.8), Brain Heart Infusion (BHI) medium, and cholesterol quantification kit were purchased from Beijing Solarbio Life Science Technology Co., Ltd. (China). Dulbecco’s modified eagle medium (DMEM) was purchased from Gibco BRL Life Technologies, Inc. (USA). Fluorescein 5-isothiocyanate (FITC) was purchased from Shanghai Beyotime Biotech Co., Ltd. (China). Syto9/Propidium Iodide (PI) bacterial staining kit was purchased from Shanghai maokangbio Biotechnology Co., Ltd. (China). *Akkermansia muciniphila* (AKK) was purchased from the General Microbiological Culture Collection Center (GMCC, China). Caco-2 cells and RAW264.7 cells were provided by the Sixth Affiliated Hospital of Sun Yat-sen University. Female C57BL/6 mice (6-8 weeks old) and female Sprague-Dawley (SD) rat (6-8 weeks old) were obtained from the Experimental Animal Center of Sun Yat-sen University. Mice were housed under specific pathogen-free (SPF) conditions at 20-30 °C with a 12 h light/dark cycle.

**Characterization**

Attenuated Total Reflectance Fourier-Transform Infrared Spectroscopy (ATR-FTIR) spectroscopy was performed using a Cary 610/670 infrared spectrometer (Agilent, USA) to verify the successful modification of alginate with L-cysteine. Scanning electron microscopy (SEM, S-4800, Hitachi, Japan) was performed to study the morphology. Energy-dispersive X-ray spectroscopy (QUANTAX EDS XFlash® 7, Bruker, Germany) and X-ray photoelectron spectroscopy (Nexsa, Thermo Fisher, USA) were used to confirm the distribution of characteristic elements of cysteine. Proton nuclear magnetic resonance (^1^H NMR) spectra were performed on Nuclear Magnetic Resonance 600 M (JNM ECZL600R, JEOL, Japan). The particle size and zeta potential of AKK samples were measured using a NanoBrook ZetaPlus (Brookhaven Inst. Corp., BI-PALS, USA). Isothermal titration calorimetry (ITC) measurements were performed using a MicroCal VP-ITC isothermal titration calorimeter (Malvern Panalytical, USA). The images of microspheres were captured, and the sizes of microspheres were measured by using an ultra-depth 3D microscope (VHX 1000C, Keyence, Japan). All tensile tests were conducted at room temperature using a universal testing machine (WD-5A, Guangzhou Experimental Instrument Factory, China).

**Preparation of AKK-COD**

AKK bacteria were grown overnight in BHI medium at 37 °C and then cultured into fresh medium at a 1:50 dilution. The culture was centrifuged at 4000 rpm for 20 min, and the pellet was washed three times with PBS to collect bacteria. A total of 1 × 10^10^ colony-forming units (CFU) of bacteria were resuspended in 1 mL Tris-HCl buffer (10 mM, pH 8.8) containing 0.1 mg mL^−1^ dopamine and 10 U mL^−1^ cholesterol oxidase (COD), followed by incubation at 37 °C with shaking for 1 h. COD-modified AKK bacteria were collected by centrifugation at 4000 rpm for 20 min and washed three times with PBS. The resulting COD modified AKK (denoted as AKK-COD) was purified by PBS washing, suspended in 1 mL physiological saline, and stored at 4 °C.

Purified AKK-COD was rinsed with PBS, fixed with glutaraldehyde, and observed by SEM. AKK was labeled with Syto9, and COD was labeled with Cy5.5 to confirm their co-localization under a fluorescence microscope. The particle size and zeta potential of AKK, AKK@PDA, and AKK-COD were measured at 1 × 10^9^ CFU mL^−1^.

**Fluorescent Labeling for COD**

COD was incubated with the dye (Cy5.5-NHS or FITC) in PBS (pH 7.2) solution at room temperature overnight in the dark. The reaction was terminated using excess Tris-HCl buffer (pH 7.2), followed by dialysis using ultrapure water three times (8 h each time) to remove unreacted free dye.

**Preparation of Ca^2+^-Crosslinked Thiol-Modified Alginate (*x*Alg-SH) Microspheres**

A 1.5% w/v sodium alginate solution (20 mL) was stirred for 3 h until completely dissolved. To activate carboxyl groups, 160 mg of EDC and 80 mg of NHS were added, followed by stirring at room temperature for 1.5 h. Subsequently, 100 mg of L-cysteine was introduced, and the reaction was conducted under nitrogen protection for 24 h. The product was dialyzed against ultrapure water (3500 MW cutoff) for 72 h (with water changing every 24 h) to remove unreacted substrates. The dialyzed product was pre-cooled and then lyophilized for 48 h to obtain lyophilized sodium alginate-cysteine (Alg-SH), which was stored at 4 °C for later use.

Microsphere preparation was carried out via a gas shearing method. A 1% w/v sodium alginate (Alg) solution or a1% w/v modified sodium alginate (Alg-SH) solution (9 mL each) were mixed with 1 mL of 1 × 10^10^ CFU FITC-labeled AKK-COD bacterial suspension to obtain AKK-COD@Alg or AKK-COD@Alg-SH mixtures at a final concentration of 1 × 10^9^ CFU mL^−1^. The mixed solution was loaded into a syringe and dropped into 100 mM CaCl_2_ solution via gas shearing (injection pump speed: 2 mL min^−1^, nitrogen flow rate: 1 L min^−1^, dropping height: 10 cm) to prepare AKK-COD@*x*Alg or AKK-COD@*x*Alg-SH microspheres. The microspheres were imaged using an ultra-depth 3D microscope, and their sizes were analyzed using ImageJ. Successful encapsulation of Syto9-labeled AKK was confirmed by fluorescence microscopy.

The encapsulation efficiency of AKK-COD in *x*Alg-SH microspheres was determined by turbidimetry using a microplate reader at 600 nm. Briefly, the initial OD₆₀₀ value of the AKK-COD suspension was recorded as OD_1_, and the volume of the suspension was recorded as V_1_. After microsphere formation, the supernatant containing unencapsulated bacteria was collected, the volume of supernatant was recorded as V_2_, and its OD₆₀₀ value was recorded as OD_2_. The encapsulation efficiency was calculated as: EE (%) = (1 − (OD_2_ × V_2_) / (OD_1_ × V_1_)) × 100. The loading capacity was estimated as: LC (CFU mg^−1^) = (Encapsulated AKK-COD amount) / (Mass of *x*Alg-SH microspheres).

**Growth Curve of AKK-COD**

AKK, AKK@PDA, and AKK-COD were diluted to equal optical density (OD) values in medium at 37 °C. The optical density at 600 nm (OD_600_) was recorded at different time points using a microplate reader (BioTek).

**pH-Responsive Release of *x*Alg-SH Microspheres**

AKK-COD@*x*Alg-SH microspheres were incubated at room temperature in simulated gastric fluid (SGF, pH = 1.5), simulated intestinal fluid (SIF, pH = 6.8), and simulated colonic fluid (SCF, pH = 7.2). Morphological changes were observed under an optical microscope, and images were recorded at 0, 0.5, and 1 h.

For release kinetics, 100 mg of AKK-COD@*x*Alg-SH microspheres were added to a 50 mL centrifuge tube containing SGF and agitated at 125 rpm in a 37 °C shaker. At 5, 10, 20, 30, and 60 min, 2 mL of supernatant was collected by centrifugation at 800 rpm for 1 min, and 2 mL of fresh SGF was replenished. The optical density at 600 nm (OD_600_) of the supernatant was measured to quantify the released AKK-COD, using a PBS-diluted AKK-COD solution (50 mL) as a baseline reference. Release curves for SIF and SCF were generated using the same protocol.

**Catalytic Activity Assay of AKK-COD**

The catalytic activity of AKK-COD was determined using a cholesterol assay kit. For microsphere-protection assessment, AKK-COD, AKK-COD@*x*Alg, and AKK-COD@*x*Alg-SH were incubated with SGF at 37 °C for 6 h in a shaker (125 rpm). After removing SGF, the microspheres were dissociated with SCF, and the supernatant containing AKK-COD was collected by centrifugation at 800 rpm for 1 min. The supernatant was then incubated with a 5 μmol mL^−1^ cholesterol solution at 37 °C and shaken at 125 rpm for 30 min. Cholesterol was measured using the assay kit to reflect cholesterol clearance efficiency.

**Gastric Acid Resistance of AKK@*x*Alg-SH**

AKK@*x*Alg and AKK@*x*Alg-SH were prepared as described. AKK (1 mL of 1 × 10^9^ CFU mL^−1^ AKK suspension), AKK@*x*Alg (100 mg), and AKK@*x*Alg-SH (100 mg) were incubated with 10 mL of SGF at 37 °C and 125 rpm for 2 h in 15 mL centrifuge tubes. The same procedure was repeated for SIF-treated groups. After removing the supernatant, microspheres were dissociated with SCF, and precipitates were removed by centrifugation at 800 rpm for 1 min. The supernatant was further centrifuged at 4000 rpm for 20 min, and the pellet was resuspended in 2 mL of PBS. AKK viability was assessed by Syto9/PI staining, and growth curves were determined by adding 1 mL of the PBS-resuspended bacteria to fresh BHI medium.

**Isothermal Titration Calorimetry (ITC)**

Solutions of 0.1% mucin, 0.3% Alg, 0.3% Alg-SH, and 1 × 10^9^ CFU mL^−1^ AKK-COD in PBS were degassed under vacuum to prevent bubbling. For mucin-Alg-SH interaction studies, the calorimetric cell was loaded with 0.1% mucin, and the titration syringe contained 0.3% Alg or Alg-SH. A constant stirring speed of 130 rpm was maintained during experiments to ensure proper mixing after each injection, with a titration volume of 200 μL and an interval of 500 s between injections to allow optimal baseline thermal compensation. For AKK-COD + Alg-SH interaction studies, the calorimetric cell was loaded with 1 × 10^9^ CFU mL^−1^ AKK-COD suspension. To investigate the effect of acidic conditions on disulfide bond formation, 0.1% mucin and 0.3% Alg-SH solutions were prepared using dilute hydrochloric acid at pH 1.5. Data were analyzed using MicroCal VP-ITC software to calculate the enthalpy change (ΔH) per injection and plot the curves.

**In Vitro Adhesion Test**

Rat small intestinal tissues were harvested, with each segment cut to a length of 5 cm. In a culture dish (10 cm diameter), each intestinal segment was longitudinally incised along the mesenteric side to expose the intestinal mucosal surface. To simulate physiological environments, 1 mL of SGF was dropped onto the mucosal surface to mimic the acidic gastric environment, while 1 mL of SIF was added to replicate the small intestinal environment. An aliquot of 1 mL of the *x*Alg or *x*Alg-SH microsphere suspension was taken and incubated with the small intestinal mucosa at 37 °C for 2 min. Subsequently, the mucosal surface was gently rinsed three times with a slow stream of PBS to remove unadhered microspheres, and the residual number of microspheres on the intestinal mucosa was observed.

All tensile tests were conducted at room temperature with a stretching speed of 30 mm min^−1^. *x*Alg or *x*Alg-SH hydrogel discs (1 mm thickness) were prepared via Ca^2+^ cross-linking. The gel discs were fixed to the base of a 1 cm-diameter syringe and clamped in the upper fixture, while SD rat small intestine mucosa (mucosa-facing upward) was fixed to another syringe base and clamped in the lower fixture. The upper fixture was adjusted to bring the gel disc into close contact with the intestinal mucosa before performing tensile tests. Each condition was tested with three independent samples, and the maximum detachment force was recorded for each trial. For mucin adhesion studies, 2% mucin (1 mL) was added to a 2 cm-diameter petri dish, fixed to the lower fixture with tape, and tested using the same procedure.

*x*Alg or *x*Alg-SH gel discs were prepared in 48-well plates by adding 200 μL of 1% Alg or Alg-SH solution and cross-linking with Ca^2+^. Cy5.5-labeled 2% mucin or 1 × 10^9^ CFU mL^−1^ Syto9-labeled AKK-COD were incubated with the gel discs at 37 °C for 30 min in the dark. After washing three times with sterile PBS, the gel discs were observed under a fluorescence microscope to assess fluorescent residue.

**In Vivo Fluorescence Imaging**

COD was pre-labeled with FITC, and AKK-COD, AKK-COD@*x*Alg, and AKK-COD@*x*Alg-SH were prepared as described above. Female C57BL/6 mice (6–8 weeks old) were gavaged with the formulations, and at 4, 8, 12, 24, and 48 h post-gavage, the small intestine and colon were harvested. Intestinal distribution of COD was visualized by in vivo fluorescence imaging. For the 48 h group, frozen sections of the upper small intestine were further analyzed via fluorescence scanning to assess the retention of COD.

**In Vitro Biocompatibility**

To evaluate the biocompatibility of AKK-COD@*x*Alg-SH, CCK-8 and Calcein-AM/PI cytotoxicity assays were used. Caco-2 or RAW264.7 cells (1 × 10^4^ cells per well) were co-cultured with COD or AKK-COD@*x*Alg-SH extracts in 96-well plates under 5% CO₂ at 37 °C. Cell proliferation was observed via fluorescence microscopy on days 1, 2, and 3, and viable cells were quantified by measuring OD_450_ after incubation with 10% CCK-8 solution for 2 h.

**Animal Grouping and Treatment**

C57BL/6 mice were randomly divided into six groups: Healthy, PBS, COD, AKK-COD, AKK-COD@*x*Alg, and AKK-COD@*x*Alg-SH. The Healthy group received a normal diet, while the other five groups were fed a high-fat (40%), high-cholesterol (1.25%), and high-sodium cholate (0.5%) diet for 2 weeks to induce hypercholesterolemia, followed by 2 weeks of treatment. The COD group was gavaged with COD (2 U d^−1^), and the AKK-COD groups with AKK at 1 × 10^9^ CFU d^−1^. Mouse body weights were recorded during the treatment period. All mice were euthanized to collect blood, intestinal contents, and organs, with liver weight measured.

**Serological Evaluation**

Serum levels of aspartate aminotransferase (AST), alanine aminotransferase (ALT), total cholesterol (TC), triglycerides (TG), high-density lipoprotein cholesterol (HDL-C), and low-density lipoprotein cholesterol (LDL-C) were measured using commercial kits according to the manufacturer’s instructions.

**In Vivo Safety Assessment**

Histological safety was evaluated via hematoxylin and eosin (HE) staining of tissue sections from the heart, liver, spleen, lung, kidney, stomach, small intestine, and colon. Histological staining was conducted by Wuhan Servicebio Technology Co., Ltd. (China).

**Evaluation of Liver Lipid Droplets**

After treatment, liver tissue sections were washed and fixed in 4% paraformaldehyde for 5 min. Following repeated rinses, the sections were stained with Oil Red O working solution at 37 °C in the dark, followed by decolorization with 60% isopropanol. Finally, Oil Red O-stained liver images were acquired using a slide scanner (ECLIPSE Ti2, NIKON Instrument Inc., Tokyo, Japan). Semi-quantitative analysis of the acquired images was conducted using ImageJ software.

**Immunohistochemical and Immunofluorescence Staining**

Jejunum samples were fixed in 4% paraformaldehyde, processed according to standard protocols for paraffin embedding. Immunohistochemical staining for NPC1L1 and ABCG5, as well as immunofluorescence staining for ZO-1 and occludin, were conducted by Wuhan Servicebio Technology Co., Ltd. (China). Semi-quantitative analysis of the acquired images was conducted using ImageJ software.

**Alcian Blue and Periodic Acid-Schiff (AB-PAS) Staining for Goblet Cells Quantification**

To assess intestinal barrier function, AB-PAS staining was performed according to the manufacturer's instructions (MST-8050, MXB Biotechnologies) to quantify the level of goblet cells in the small intestinal mucosa. Stained section images were acquired using a digital slide scanner and subsequently analyzed with ImageJ.

**16S rRNA Sequencing**

16S rRNA sequencing was used to deeply analyze the bacterial composition in mouse small intestinal contents to determine whether and how AKK-COD@*x*Alg-SH influences the small intestinal microbiota. Small intestinal contents from each mouse were collected post-mortem and stored at −80 °C. Total DNA was extracted from the contents, and sequencing was performed on an Illumina HiSeq platform by targeting the hypervariable V3-V4 region of the bacterial 16S rRNA gene. Data analysis was conducted on the Majorbio Biocloud platform, and microbial composition was determined using the Illumina HiSeq platform. Operational taxonomic units (OTUs) were clustered using UPARSE (version 7.1). Alpha diversity was quantified using the Ace and Chao1 indices, while Beta diversity was analyzed via principal component analysis (PCA). Microbial composition was assessed at the phylum and class levels, and linear discriminant analysis (LDA) was performed to identify microbial communities or species contributing significantly to sample differentiation based on taxonomic composition.

**Ethical Approval Statement**

All experiments complied with international guidelines, and animal studies were approved by the Institutional Animal Care and Use Committee of Sun Yat-sen University (License No: 2024000290).

**Statistical Analysis**

All statistical analyses were performed using Origin 2021 software (Origin Lab Incorporation, Northampton, USA). Experimental data are presented as the mean ± standard deviation (SD). To assess statistical differences among multiple groups, one-way analysis of variance (ANOVA) with Tukey’s multiple comparisons was employed. Statistical significance was denoted as follows: * indicates *p* < 0.05, ** indicates *p* < 0.01, and *** indicates *p* < 0.001.


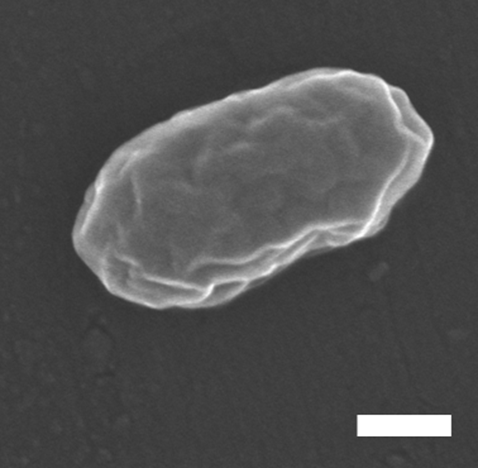


**Figure S1.** SEM image of AKK. Scale bar: 200 nm.


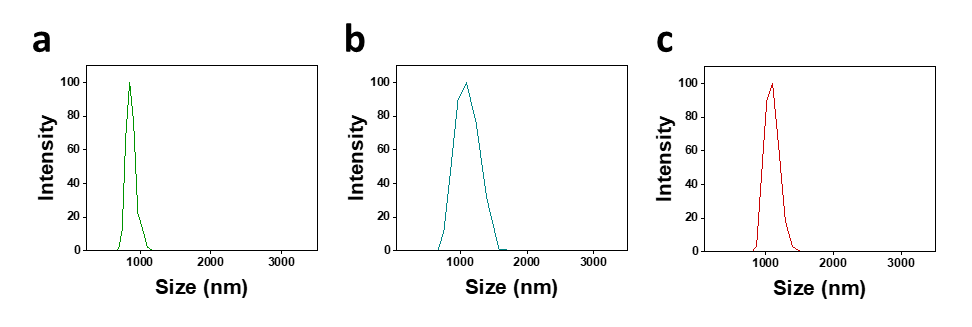


**Figure S2.** Particle size distribution curves for AKK (a), AKK@PDA (b), and AKK-COD (c).


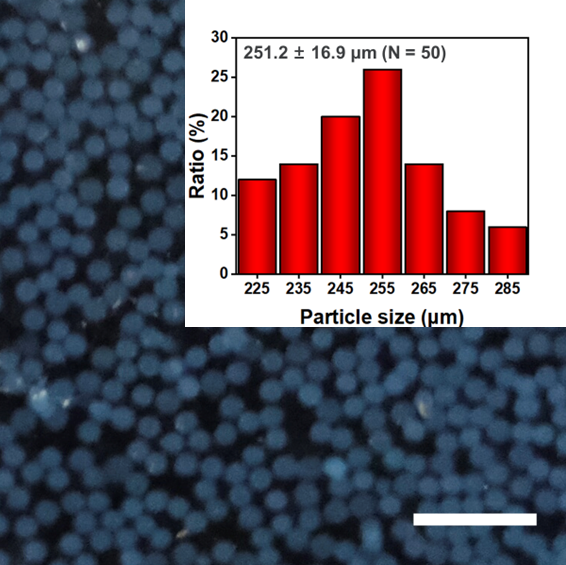


**Figure S3.** Digital photo and particle size distribution (inset) of *x*Alg-SH microspheres. Scale bar: 2 mm.


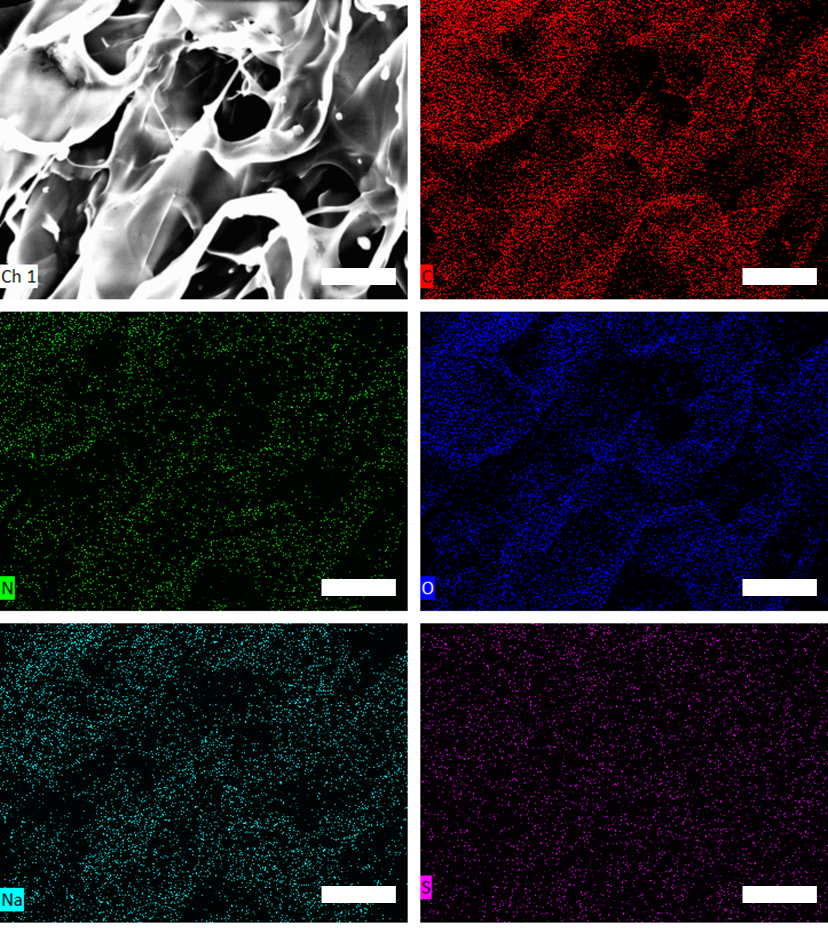


**Figure S4.** SEM-EDS imaging result of Alg-SH. Scale bars: 30 μm.

**Figure S5.** EDS elemental spectrum chart result of Alg-SH.


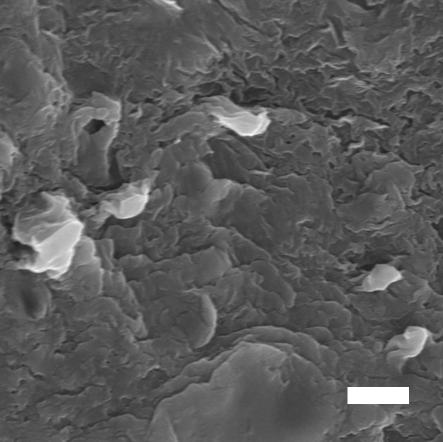


**Figure S6.** SEM image for the interior of AKK-COD@*x*Alg-SH. Scale bar: 1 μm.

**Figure S7.** Catalytic activity of AKK-COD over 7 days following SGF exposure.


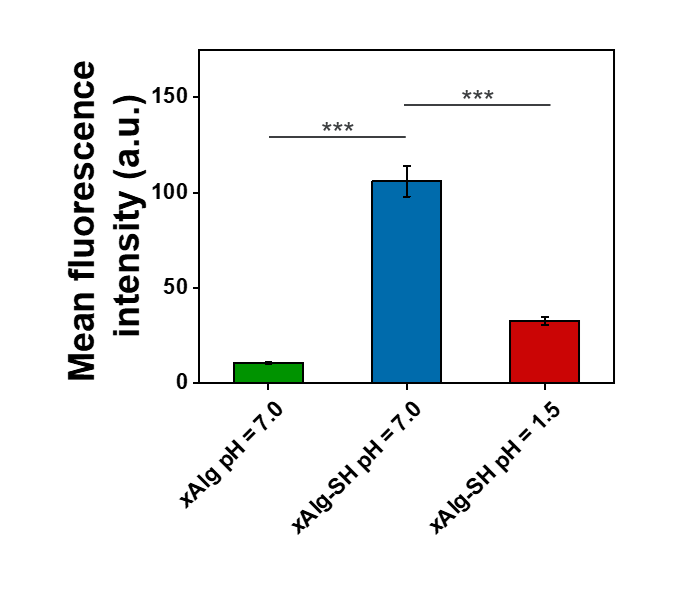


**Figure S8.** Quantitative analysis of mucin retention on the *x*Alg or *x*Alg-SH membranes (*n* = 3 independent samples; the data are presented as mean ± SD; ANOVA followed by Tukey’s multiple comparisons; *** adjusted *p*  < 0.001).


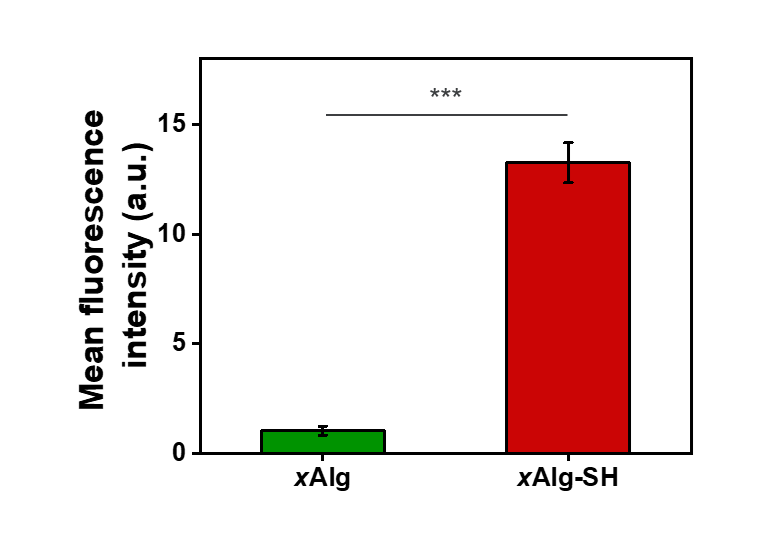


**Figure S9.** Quantitative analysis of AKK retention on the *x*Alg or *x*Alg-SH membranes (*n* = 3 independent samples; the data are presented as mean ± SD; ANOVA followed by Tukey’s multiple comparisons; *** adjusted *p*  < 0.001).


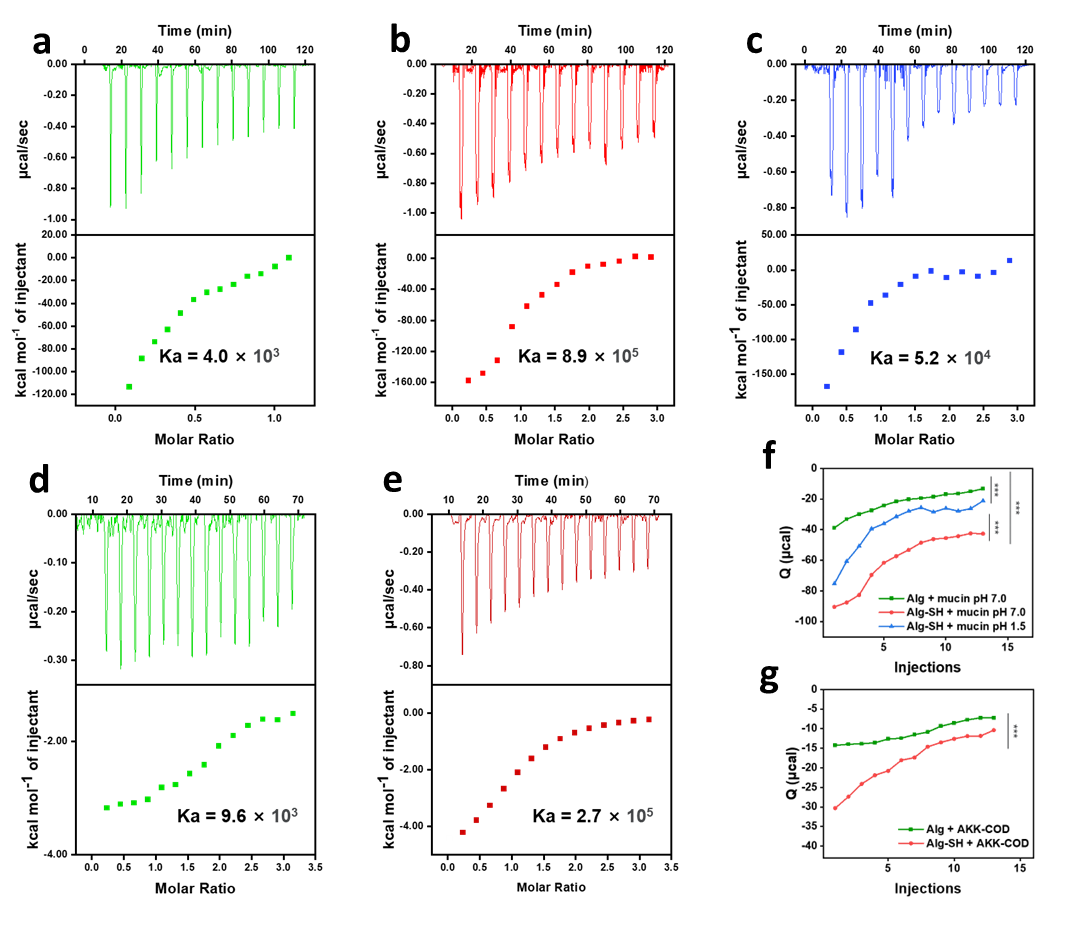


**Figure S10.** ITC titration curves of Alg + mucin (a), Alg-SH + mucin (b), Alg-SH + mucin at pH 1.5 condition (c), Alg + AKK-COD (d), and Alg-SH + AKK-COD (e). f) ITC results of interactions between mucin and Alg-SH or Alg. g) ITC results of interactions between AKK-COD and Alg-SH or Alg.


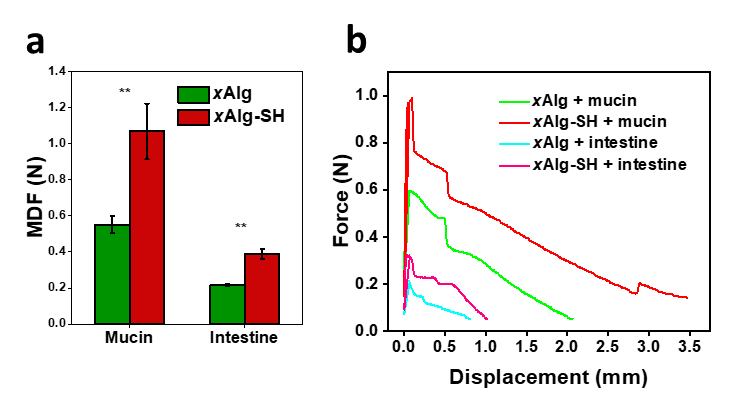


**Figure S11.** a) Adhesion force measurements of *x*Alg-SH and *x*Alg with mucin or SD rat small intestine (*n* = 3 independent samples; the data are presented as mean ± SD; ** *p*  < 0.01). b) Adhesion force as a function of displacement for *x*Alg or *x*Alg-SH hydrogel discs against mucin or SD rat intestinal mucosa.


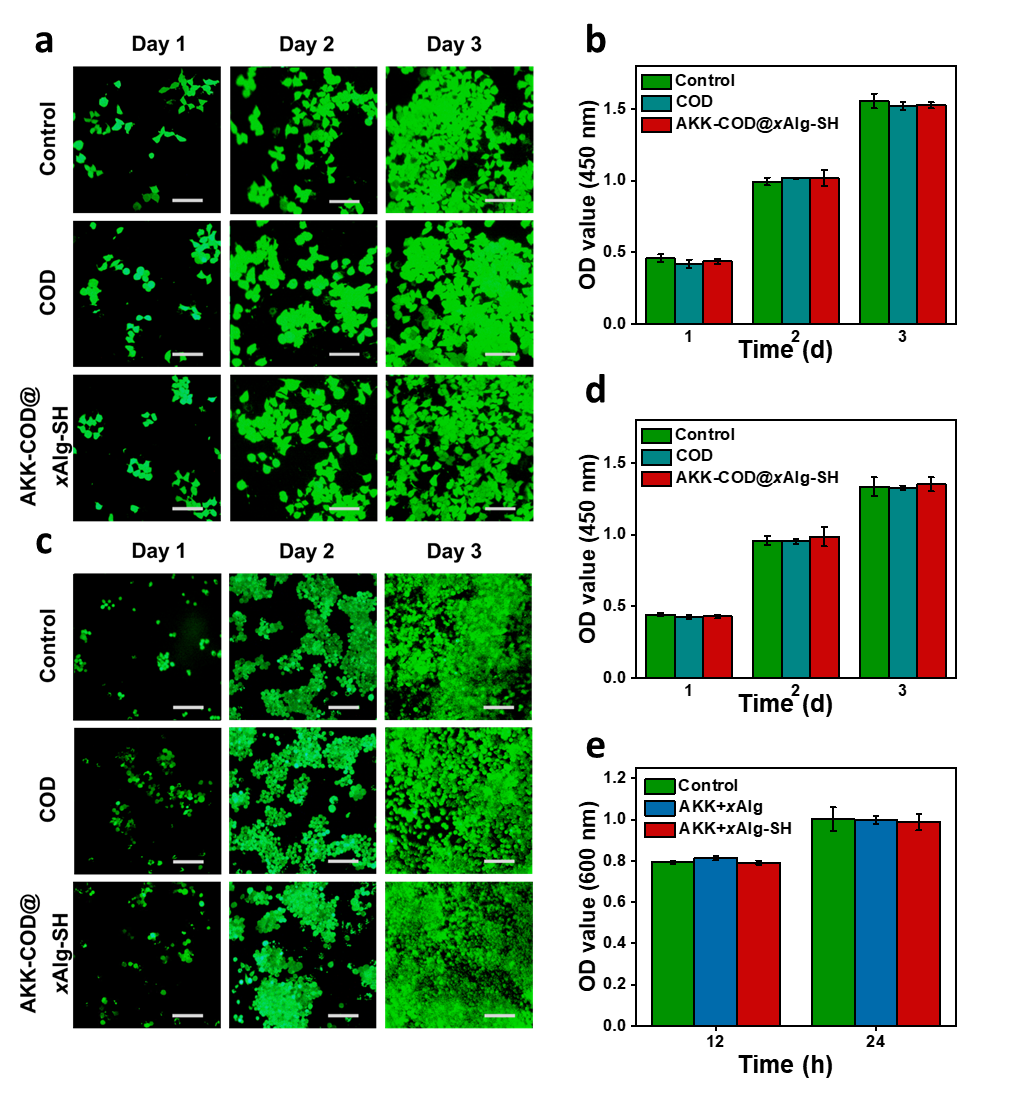


**Figure S12.** Biocompability of AKK-COD@*x*Alg-SH. a,b) Live/dead staining (a) and CCK-8 assay (b) for Caco-2 cells. Scale bars: 100 μm. c,d) Live/dead staining (c) and CCK-8 assay (d) for RAW264.7 cells. Scale bars: 100 μm. e) AKK growth after co-culture with *x*Alg or *x*Alg-SH microspheres (*n* = 3 independent samples; the data are presented as mean ± SD).


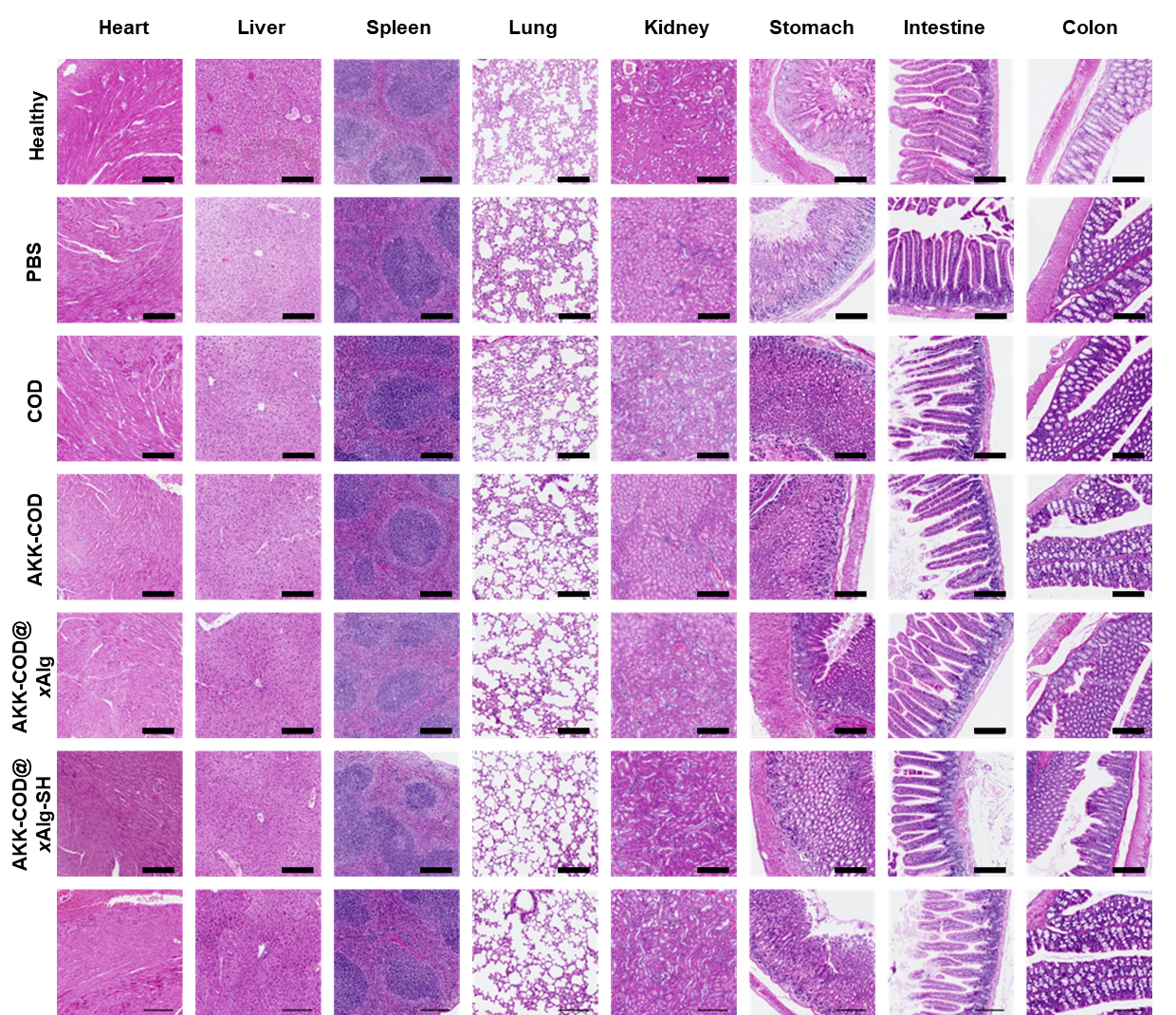


**Figure S13.** HE staining for organ assessment after treatment. Scale bars: 200 μm.


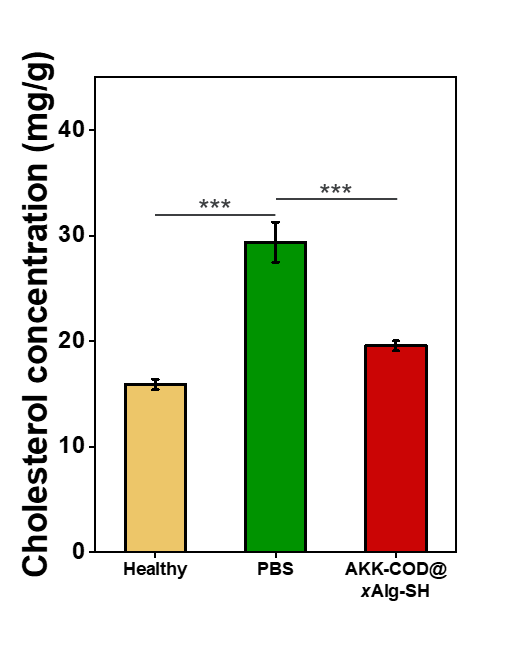


**Figure S14.** Feces cholesterol concentration among different groups, suggesting the cholesterol in feces is converted by AKK-COD (*n* = 3 independent samples; the data are presented as mean ± SD; ANOVA followed by Tukey’s multiple comparisons; *** adjusted *p*  < 0.001).


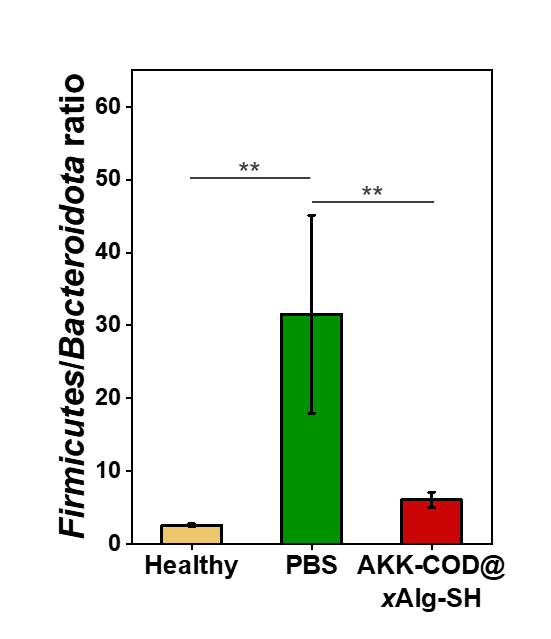


**Figure S15.** *Firmicutes/Bacteroidota* ratio among groups determined by 16S rRNA sequencing (*n* = 3 independent samples; the data are presented as mean ± SD; ANOVA followed by Tukey’s multiple comparisons; ** adjusted *p* < 0.01).


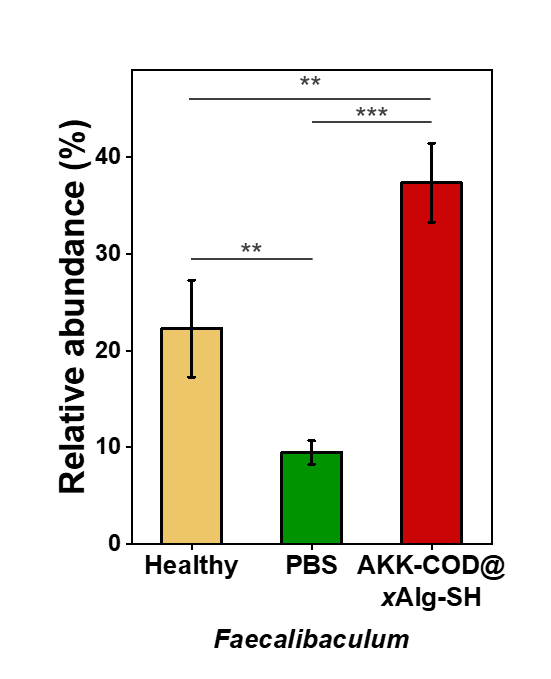


**Figure S16.** Relative abundance of bacteria determined by 16S rRNA sequencing (*n* = 3 independent samples; the data are presented as mean ± SD; ANOVA followed by Tukey’s multiple comparisons; ** adjusted *p* < 0.01, *** adjusted *p*  < 0.001).
